# Metformin reduces basal subpopulation and attenuates mammary epithelial cell stemness in FVB/N mice

**DOI:** 10.3389/fcell.2024.1427395

**Published:** 2024-07-11

**Authors:** Minghui Shan, Qiong Cheng, Amanda B. Parris, Lingfei Kong, Xiaohe Yang, Yujie Shi

**Affiliations:** ^1^ Department of Pathology, People’s Hospital of Zhengzhou University, Zhengzhou, Hena, China; ^2^ Academy of Medical Sciences, Zhengzhou University, Zhengzhou, Henan, China; ^3^ Biomedical/Biotechnology Research Institute, Department of Biological and Biomedical Sciences, North Carolina Research Campus, North Carolina Central University, Kannapolis, NC, United States

**Keywords:** metformin, basal epithelial cells, mammary stem cells, receptor tyrosine kinase, estrogen receptor, breast cancer prevention

## Abstract

Metformin shows promise in breast cancer prevention, but its underlying mechanisms remain unclear. This study investigated the impact of metformin on the repopulation dynamics of mammary epithelial cells (MECs) and the signaling pathways in non-tumorigenic FVB/N mice. This study aimed to enhance our understanding of the role of metformin in reducing the susceptibility of MECs in premalignant tissues to oncogenic factors. In this study, female mice were administered 200 mg/kg/day of metformin via intraperitoneal (i.p.) injection from 8 to 18 weeks of age. After this treatment period, morphogenesis, flow cytometry, analyses of MEC stemness, and RNA sequencing were performed. The study findings indicated that metformin treatment in adult mice reduced mammary gland proliferation, as demonstrated by decreased Ki67+ cells and lateral bud formation. Additionally, metformin significantly reduced both basal and mammary repopulating unit subpopulations, indicating an impact on mammary epithelial cell repopulation. Mammosphere, colony-forming cell, and 3D culture assays revealed that metformin adversely affected mammary epithelial cell stemness. Furthermore, metformin downregulated signaling in key pathways including AMPK/mTOR, MAPK/Erk, PI3K/Akt, and ER, which contribute to its inhibitory effects on mammary proliferation and stemness. Transcriptome analysis with RNA sequencing indicated that metformin induced significant downregulation of genes involved in multiple critical pathways. KEGG-based pathway analysis indicated that genes in PI3K/Akt, focal adhesion, ECM-receptor, small cell lung cancer and immune-modulation pathways were among the top groups of differentially regulated genes. In summary, our research demonstrates that metformin inhibits MEC proliferation and stemness, accompanied by the downregulation of intrinsic signaling. These insights suggest that the regulatory effects of metformin on premalignant mammary tissues could potentially delay or prevent the onset of breast cancer, offering a promising avenue for developing new preventive strategies.

## Introduction

The prevention and treatment of breast cancer present significant challenges, necessitating innovative approaches for its management and treatment. The anti-diabetic drug metformin has emerged as an anti-cancer agent (Pernicova and Korbonits, 2014; Saini and Yang, 2018; De and Kuppusamy, 2020; Zhao et al., 2020), which is supported by studies on various cancer types, including breast, lung, prostate and ovarian cancers (Col et al., 2012; Ahn et al., 2020; Han et al., 2023; Lu et al., 2023). Epidemiological studies suggest a potential link between metformin use in patients with diabetes and a decreased risk of breast cancer (Col et al., 2012). Preclinical studies largely support metformin-associated anti-cancer effects (De and Kuppusamy, 2020). The associated mechanisms include systemic reduction in insulin levels, modulation of metabolic pathways, and direct modulation of critical pathways, such as activation of the AMP-activated protein kinase (AMPK) pathway and inhibition of the insulin/IGF-1 pathway, which are involved in the regulation of metabolism and proliferation (Pernicova and Korbonits, 2014). Inhibition of cancer stem cells may also play a critical role in metformin-induced anti-cancer activities (Saini and Yang, 2018). However, clinical trials investigating metformin’s anti-cancer effects as a therapeutic agent for breast cancer have yielded inconsistent outcomes (Cejuela et al., 2022). The potential use of metformin as a preventive agent carries significant implications. Differentiating between its therapeutic and preventive roles is essential in cancer management. Epidemiological evidence indicating a reduced incidence of cancer among diabetes patients treated with metformin has stimulated interest in its preventive capabilities.

Unlike the majority of preclinical research that concentrates on breast cancer cell lines or tumor models (De and Kuppusamy, 2020), investigations into the impact of metformin on breast tissues before tumorigenesis or on non-tumor tissues could yield valuable insights into its preventative mechanisms (Pernicova and Korbonits, 2014; Shehata et al., 2019). This study aimed to investigate the potential of metformin in breast cancer prevention by assessing its impact on intrinsic pathways within the mammary tissue hierarchy using a non-tumorigenic mouse model. This study aimed to elucidate the repopulation dynamics of mammary epithelial cells (MECs) in non-tumor-bearing mice, reducing their vulnerability to oncogenic factors, by focusing on their repopulation dynamics. The significance of this investigation lies in its innovative approach to cancer prevention, leveraging a well-established drug to potentially disrupt early cellular processes that predispose an individual to malignancy. Understanding the role of metformin in MEC behavior could pave the way for novel preventative strategies for breast cancer, highlighting the importance of reevaluating existing medications for new therapeutic applications.

Regulation of mammary stem cells (MaSCs) and MEC progenitor cells play a critical role in mammary gland development and homeostasis (Richert et al., 2000; Visvader and Smith, 2011). Increasing evidence has underscored the close relationship between the modulation of MaSC and MEC stemness and breast cancer risk or prevention (Siwko et al., 2008; Tiede and Kang, 2011; Wang et al., 2014). Recent advances have resulted in the development of various technologies for MEC/MaSC subpopulation profiling and functional characterization. Alterations in MEC subpopulations, including luminal, basal, and mammary repopulating units (MRUs), which serve as surrogate markers of MaSC enrichment, indicate MEC reprogramming in response to various factors (Stingl, 2009; Visvader, 2009). Our previous studies demonstrate that biguanide drugs induced inhibition of mammary tumors in the FVB/N-MMTV-erbB2 transgenic mice and involves selective inhibition of basal MEC subpopulations and MaSC stemness in premalignant tissues (Liu et al., 2011; Zhu et al., 2014; Guo et al., 2017; Parris et al., 2017). In a recent study using a normal C57BL/6 mouse model, metformin intake selectively reduced the total cell number and progenitor capacity of the hormone receptor-positive (HR+) luminal population in mammary tissues, correlating with reduced mitochondrial respiration and DNA damage (Shehata et al., 2019). While previous studies demonstrated metformin-induced changes in MEC subpopulations and metabolism in normal and premalignant mammary tissues, the specific MEC subpopulations targeted in these studies differ. Considering that our previous studies utilized the MMTV-erbB2 transgenic mouse model (Liu et al., 2011; Zhu et al., 2014; Guo et al., 2017; Parris et al., 2017), further characterization is needed to understand the interactions between metformin and intrinsic pathways in mammary tissues of non-tumor models with similar genetic backgrounds, as well as the modulated signaling within the molecular network.

In this study, we investigated metformin-induced alterations in MEC populations and stemness, combining phenotypic analyses with signal transduction studies in FVB/N mice, the parental strain of the FVB/N-MMTV-erbB2 transgenic mice. Our findings demonstrate that metformin induces MEC reprogramming and diminishes the dynamics of epithelial stemness in normal mammary tissues, along with inhibiting the intrinsic crosstalk between receptor tyrosine kinase (RTK) and ER signaling pathways involved in cellular microenvironment modulation. Studying the complex interactions between the mammary cellular architecture will enhance our understanding of the role of metformin in the modulation of mammary development.

## Materials and methods

### Antibodies and reagents

Metformin was obtained from Sigma-Aldrich. p-Akt (Ser473) (4060), p-Erk1/2 (Thr202/Tyr204) (9101), Cyclin D1 (2978), p-ERα (Ser118) (2511), p-ERα (Ser167) (5587), c-Myc (5605), p-AMPKα (Thy172) (2535), AMPKα (2532), p-mTOR (Ser2448) (5536), mTOR (2983), p-STAT3 (Tyr705) (9145), S6 (2217), p-S6 (Thr389) (9205), IRS1 (3407), p-IRS1 (Ser612) (3203) and β-catenin (8480) primary antibodies were sourced from Cell Signaling Technologies. Primary antibodies against Akt1 (sc-5298), Erk2 (sc-1647), ERα (sc-8002), STAT3 (sc-483), and β-actin (sc-47778) were purchased from Santa Cruz Biotechnology. The Active β-catenin (05-665) antibody was obtained from EMD Millipore. Anti-rabbit (7074) and anti-mouse (7076) HRP-linked secondary antibodies were from Cell Signaling Technologies. The Ki67 (PA5-19462) antibody was purchased from Invitrogen and the secondary anti-rabbit antibody for immunohistochemistry was part of the Vecta-Stain ABC kit (Vector Labs). Flow cytometry antibodies against CD16/32 (553141), CD49f (555735), CD24 (553260), and CD61 (553345) were purchased from BD Biosciences, and those against CD31 (102508), CD45 (103106), and Ter-119 (116208) were purchased from BioLegend.

### Animal handling and metformin administration

Female FVB/N mice were procured from Jackson Laboratory and housed under a consistent 12-h light-dark cycle on an estrogen-free AIN-93G diet (Synergy Bio). At 8 weeks of age, mice were randomly assigned to each of the control or metformin-treated groups. Metformin was administered daily at a dose of 200 mg/kg in 100 μL via intraperitoneal injection, while a saline solution served as the vehicle control. The dose selection was based on previous studies in mouse models that used metformin doses ranging from 100–250 mg/kg (Wu et al., 2012; Chen et al., 2017; Howell et al., 2017). Treatment was initiated at 8 weeks of age and continued for 10 weeks. At the endpoint, mice were euthanized by CO_2_ asphyxiation followed by cervical dislocation to verify animal death before tissue collection. Inguinal (#4) mammary glands were collected from mice in each group for primary mammary epithelial cell isolation from fresh tissue or snap-frozen for protein lysate/RNA extraction. For each group, 15 mice were used for wholemount/histopathology, Western blot analysis, RNA extraction, mammosphere and colony-forming cell (CFC) assays, and flow cytometry analysis as specified in individual assays. All animal experiments were approved by the Institutional Animal Care and Use Committee.

### Mammary gland wholemount preparation and analysis

Inguinal mammary glands from both control and treated mice were collected and fixed overnight in Carnoy’s solution. Following rehydration, the glands were stained with carmine alum staining solution. After staining, the glands were dehydrated, cleared with xylene, and mounted using Permount (Zhu et al., 2014). Ductal densities of whole mounts from five mice per group were examined and imaged using a Leica Microscope.

### Immunohistochemistry (IHC) staining

Formalin-fixed mammary glands from 18-week-old mice were processed for routine paraffin embedding and tissue sectioning. IHC was performed using the VECTASTAIN Elite ABC kit (Vector Laboratories) following the manufacturer’s instructions. The slides were first incubated with 10% goat serum to block non-specific binding and then incubated with a primary antibody Ki67 (1:5,000) at 4°C overnight, followed by incubation with a biotinylated secondary anti-rabbit antibody and ABC reagent. Diaminobenzidine (DAB) was used for color development, and the slides were counterstained with hematoxylin (Parris et al., 2017). Imaging and documentation were performed using a Leica Microscope.

### Isolation of primary mammary epithelial cells and flow cytometry analysis

Freshly collected mammary glands from 18-week-old mice were dissected, minced, and enzymatically dissociated overnight using a gentle collagenase and hyaluronidase kit (StemCell Technologies) according to the manufacturer’s protocol. After digestion with trypsin-EDTA and Dispase/DNase I, the resulting cell suspension was strained through a 40 - µm mesh filter to obtain a single cell suspension for flow cytometry analysis or mammosphere and CFC assays. For flow cytometry, primary MECs were incubated with anti-CD16/32 antibody to block non-specific binding to Fc receptors, and then stained with fluorescent antibodies targeting CD49f, CD24, CD61, and lineage markers (CD31, CD45, and Ter119), as previously described (Zhu et al., 2014; Parris et al., 2017). Flow cytometric analysis was performed using a BD FACSCalibur Flow Cytometer, as described previously (Zhu et al., 2014; Parris et al., 2017). With appropriate gating, lineage-negative cells exhibiting different patterns of CD24 and CD49f staining were used to identify luminal, basal, stromal, and MRU (mammary repopulating units) subpopulations. CD61 and CD49f were used as markers to identify the subpopulations enriched in luminal progenitor cells (Lo et al., 2012). FlowJo analysis software was utilized for gating and quantifying individual cell populations.

### Mammosphere assay

Primary MECs isolated from mammary tissues were cultured in ultra-low-attachment 24-well plates at a density of 2.5 × 10^4^ cells per well under specific conditions conducive to sphere formation. The culture medium consisted of EpiCult-B mouse media (Stemcell Technologies) supplemented with 10 μg/mL insulin (Sigma), 1 μg/mL hydrocortisone (Sigma), 1× B-27 (Thermo Scientific), 20 ng/mL EGF (Stemcell Technologies), 20 ng/mL basic FGF (Stemcell Technologies), 4 μg/mL heparin (Stemcell Technologies), and 50 μg/mL gentamycin. After 7 days, spheres with a diameter greater than 30 μm were counted and imaged. Primary spheres were then dissociated into single-cell suspensions and inoculated into new plates at a density of 1 × 10^3^ cells/well under identical conditions to assess the secondary sphere-forming efficiency (Zhu et al., 2014; Parris et al., 2017). Both primary and secondary sphere assays were performed in triplicate.

### 3D culture assay

Primary MECs from control and metformin-treated FVB/N mice were seeded at a density of 1.5 × 10^4^ cells per well in Matrigel-coated 48-well plates for 7 days, following procedures described in a previous report (Parris et al., 2017). Colonies from the 3D cultures were fixed with 4% paraformaldehyde for 6 h and then stained overnight with crystal violet. The colonies were imaged to analyze anchorage-independent cell growth (Parris et al., 2017). These assays were performed in triplicate.

### Colony-forming cell (CFC) assay

Isolated primary MECs were seeded at a density of 4 × 10^3^ cells per plate in 60-mm plates and cultured in supplemented EpiCult-B mouse medium for 7 days. Following incubation, the primary cells were washed, fixed with methanol/acetone (1:1), and stained with Wright’s Giemsa solution (Parris et al., 2017). Stained colonies were imaged using a Leica Microscope, counted, and analyzed. These assays were performed in triplicate.

### Western blot analysis

Mammary tissues were homogenized in the lysis buffer containing 50 mM Tris/pH 8.0, 5 mM EDTA, 150 mM NaCl, 1% NP-40, 1 mM phenylmethylsulfonyl fluoride, and 30 μL of protease inhibitor cocktail (ThermoFisher Scientific) per ml, using a tissue homogenizer for protein lysate preparation (Zhu et al., 2014). The protein concentration of the resulting supernatant was quantified using a BCA protein assay kit (Pierce). Subsequently, 50 μg of protein lysate from each sample was separated by SDS-PAGE, followed by transferring to PVDF membranes. The membranes were incubated with 5% milk in TBST buffer to block non-specific binding. Subsequently, the blots were probed with the specified primary antibodies at a dilution of 1:1000-2000 (Cell Signaling Technologies) overnight. After washing, the membranes were incubated with horseradish peroxidase-labeled secondary antibodies at 1:4000 in TBST/5% milk for 1 h, followed by chemiluminescent signal development using SuperSignal West Pico ECL reagent (Thermo Fisher Scientific). The specific protein bands were visualized and documented. The relative signal of each marker was quantified with densitometry analysis and normalized with loading control, which is summarized in Supplementary Figure S1.

### Gene expression profiling with RNA sequencing and bioinformatic analysis

The fourth pair of mammary glands, with lymph nodes removed, were collected at the endpoint and snap-frozen in liquid nitrogen. Total RNA was extracted using TRIzol (Invitrogen) according to the manufacturer’s instructions. Three RNA samples of individual mice from each of the control and metformin-treated groups were processed for further analysis. RNA sequencing and analysis of the samples were performed by the genomics service provider Biomarker Technologies (BMKGENE). RNA quality was assessed with an Agilent 2100 Bioanalyzer before library preparation. The qualified cDNA library was proceeded with RNA sequencing using a high-throughput sequencing platform in PE150 mode. Raw data were filtered by removal of adapter sequences and low-quality reads, followed by mapping clean data to the *Mus musculus* genome reference GRCm38 with efficient mapping ratios of 95.49%–98.06%. Gene expression levels were quantified with FPKM (Fragments Per Kilobase of transcript per Million fragments mapped) calculation. The differentially expressed genes (DEGs) were analyzed with DESeq2 (Subramanian et al., 2005). Criteria for differentially expressed genes were set as fold change (FC) ≥ 1.5 and *p*-value <0.01. DEGs induced by metformin were processed and presented in a volcano plot. Hierarchical clustering of DEGs was presented in a heatmap. For GO enrichment analysis, the DEGs were mapped to GO terms covering biological process, molecular function, and cellular component using ClusterProfiler. For KEGG (Kyoto Encyclopedia of Genes and Genome) pathway analysis, the DEGs were annotated and mapped to KEGG pathways to determine significant pathways impacted by the expression changes. Visualization of the interaction network among the top 20 KEGG pathways identified above were performed using the ClueGO (v2.5.10) plugin in Cytoscape (v3.9.1).

### Statistical analysis

Other than the bioinformatics analysis, data comparisons between the control and metformin-treated groups were statistically analyzed using Student’s t-test. GraphPad Prism software was employed for the analyses, with significance set at *p* ≤ 0.05. The results are presented as the mean ± SEM, based on at least three independent experimental replicates.

## Results

### Metformin suppresses mammary epithelial cell proliferation and modifies mammary morphogenesis

**FIGURE 1:**
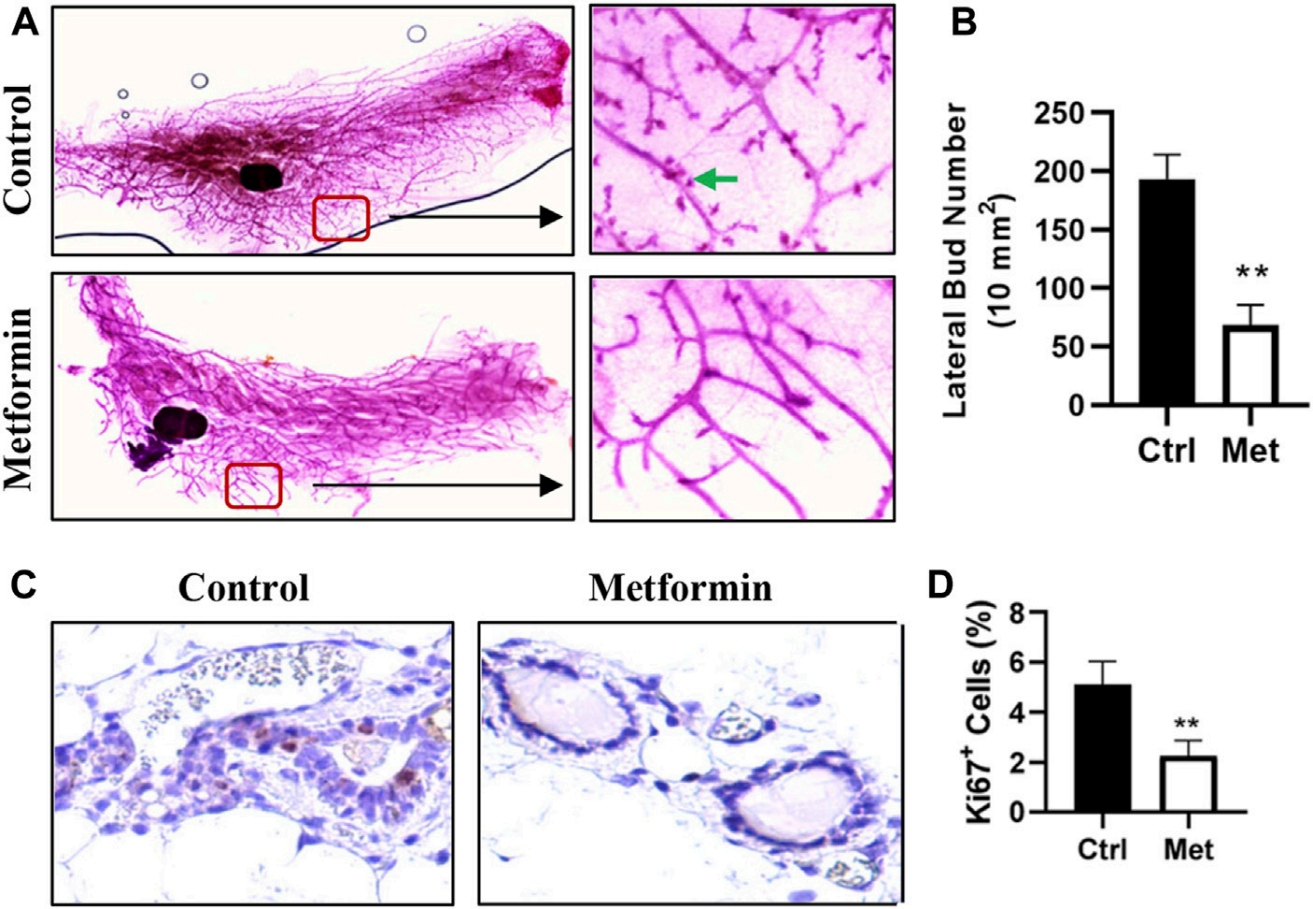
Metformin inhibits mammary epithelial cell proliferation in FVB/N mice. Female mice were exposed to metformin at 200 mg/kg/day for 10 weeks. Mammary glands were collected for wholemount and histopathology analysis. **(A, B)**. Representative mammary wholemounts of control and metformin-treated mice. Metformin-treated mammary glands display reduced mammary epithelial cell density and lateral buds/branches (arrows in green) **(A)**. Lateral bud numbers per 10 mm^2^ based on five mice of each group were quantified in **(B)**; **(C, D)**. Ki67 expression in mammary epithelial cells by immunohistochemistry. Ki67+ cells were detected with DAB as chromogen (dark brown color). **(C)** Representative images of Ki67 staining of mammary tissues. The percentage of Ki67+ mammary epithelial cells was quantified in **(D)**. (**p < 0.01).

To assess the impact of metformin on MECs in normal mammary tissues, we evaluated metformin-induced morphological changes by performing wholemount analysis of inguinal mammary glands after 10 weeks of metformin treatment. A comparison of wholemounts from the two groups revealed a noticeable reduction in mammary ductal growth and epithelial cell density in the metformin-treated group (Figure 1). Notably, metformin treatment significantly decreased the number of lateral buds and alveolar structures, indicating an inhibitory effect (Figures 1A, B). Immunohistochemical staining further demonstrated a significant reduction in the number of Ki67+ MECs in metformin-treated mammary tissues (Figures 1C, D). These findings indicate that metformin not only suppresses the proliferation rate of MECs but also inhibits lateral bud formation and branching in mammary tissues.

### Metformin induces mammary epithelial cell repopulation with significant inhibition of basal, MRU, and CD61+/CD49f+ subpopulations

**FIGURE 2:**
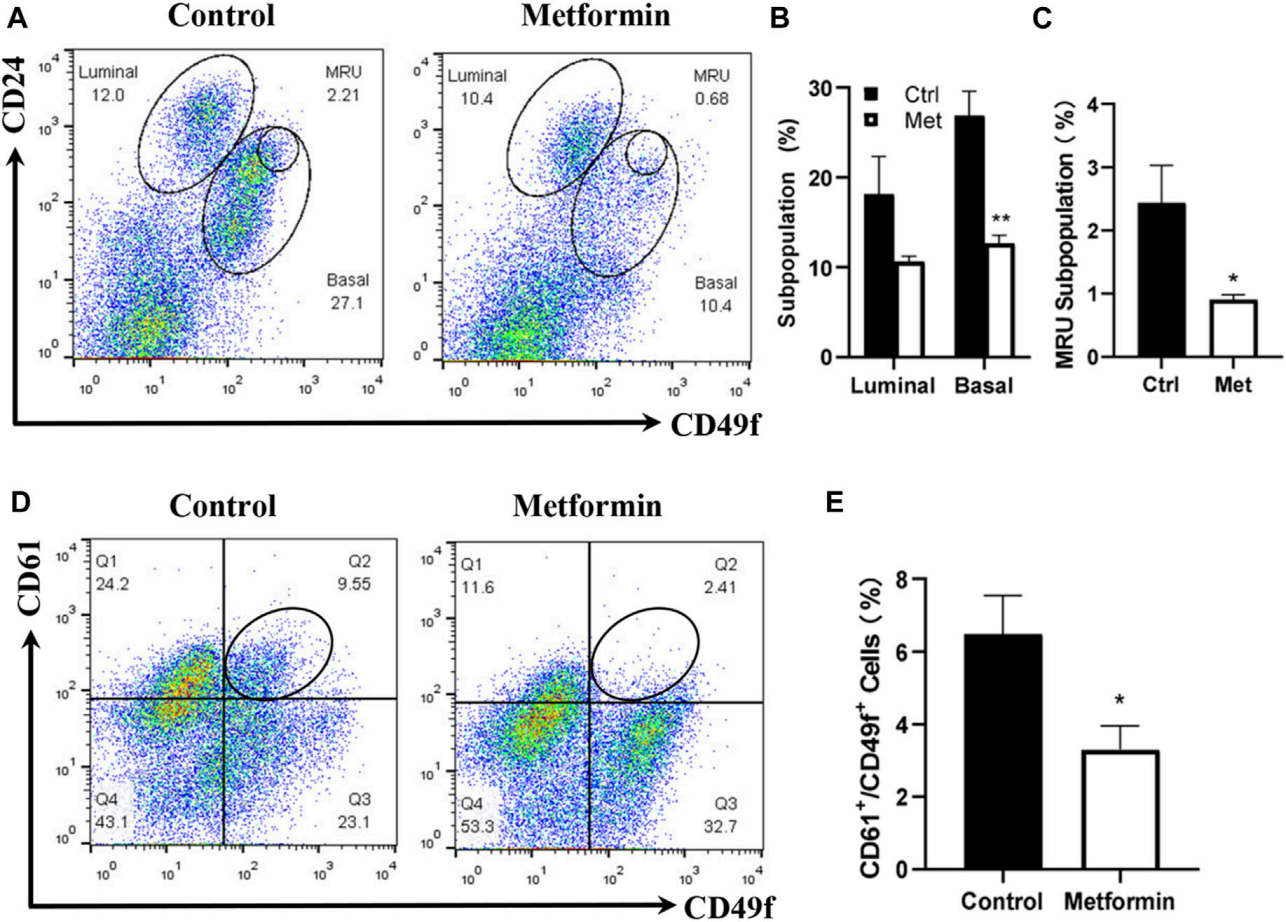
Metformin induces the repopulation of mammary epithelial cells with a significant reduction of basal and MRU subpopulations. Mammary cells isolated from control and metformin-treated mice were analyzed for different MEC subpopulations with flow cytometry. CD24 and CD49f were used to detect the relative composition of luminal, basal, and MRU subpopulations **(A–C)**. CD61 and CD49f were used to detect luminal progenitor cells (CD61+/CD49f+) enriched subpopulation **(D–E)**. **(A)** Representative plots of flow cytometry analysis based on CD24 and CD49f for luminal, basal, and MRU (mammary repopulating units) subpopulations. **(B, C)** Percentages of luminal/basal **(B)** and MRU **(C)** subpopulations from four replicates of each group were statistically analyzed; **(D)** Representative plots from the detection of CD61+/CD49f+ cells in mammary tissues of control and metformin-treated mice. **(E)** Quantification of CD61+/CD49f+ cells of each group as in B and C based on data obtained from four replicates. (*p < 0.05; **p < 0.01).

Prior studies by us and others suggested that metformin induces MEC repopulation but that the major targeted subpopulations differed (Zhu et al., 2014; Shehata et al., 2019). To examine the effect of metformin on MEC subpopulations in FVB/N mice, we characterized the MECs of metformin-treated mice using CD24/CD49f-based flow cytometry, which detects the relative composition of luminal, basal, and mammary repopulating units (MRUs) and stromal subpopulations. Our analysis revealed distinct differences in the MEC composition between metformin-treated tissues and controls (Figures 2A, B). Although metformin induced a noticeable decrease in the luminal subpopulation, the difference was not statistically significant. In contrast, basal and MRU subpopulations enriched with mammary stem cells were significantly inhibited in the metformin group (Figure 2C). This suggests that metformin induces MEC repopulation and downregulates the basal subpopulation, contributing to its modulatory effects on mammary tissues in FVB/N mice.

CD61 is a surrogate marker for luminal progenitor cells (Vaillant et al., 2008), and the CD61+/CD49+ subpopulation is enriched in luminal progenitor and tumor-initiating cells in FVB/N-MMTV-erbB2 mice (Lo et al., 2012). We previously demonstrated that metformin significantly reduced CD61+/CD49+ cells in MMTV-erbB2 mice (Zhu et al., 2014). In this study, the CD61+/CD49f+ subpopulation in the metformin-treated mammary tissues of FVB/N mice also exhibited a significant decrease compared to that in the controls (Figures 2D, E). Results from both CD24/CD49f and CD61/CD49f profiles suggest that metformin induces a repopulation of MECs in FVB/N mice, significantly affecting subpopulations enriched with mammary stem and progenitor cells.

### Metformin attenuates mammary epithelial cell stemness in FVB/N mice

**FIGURE 3:**
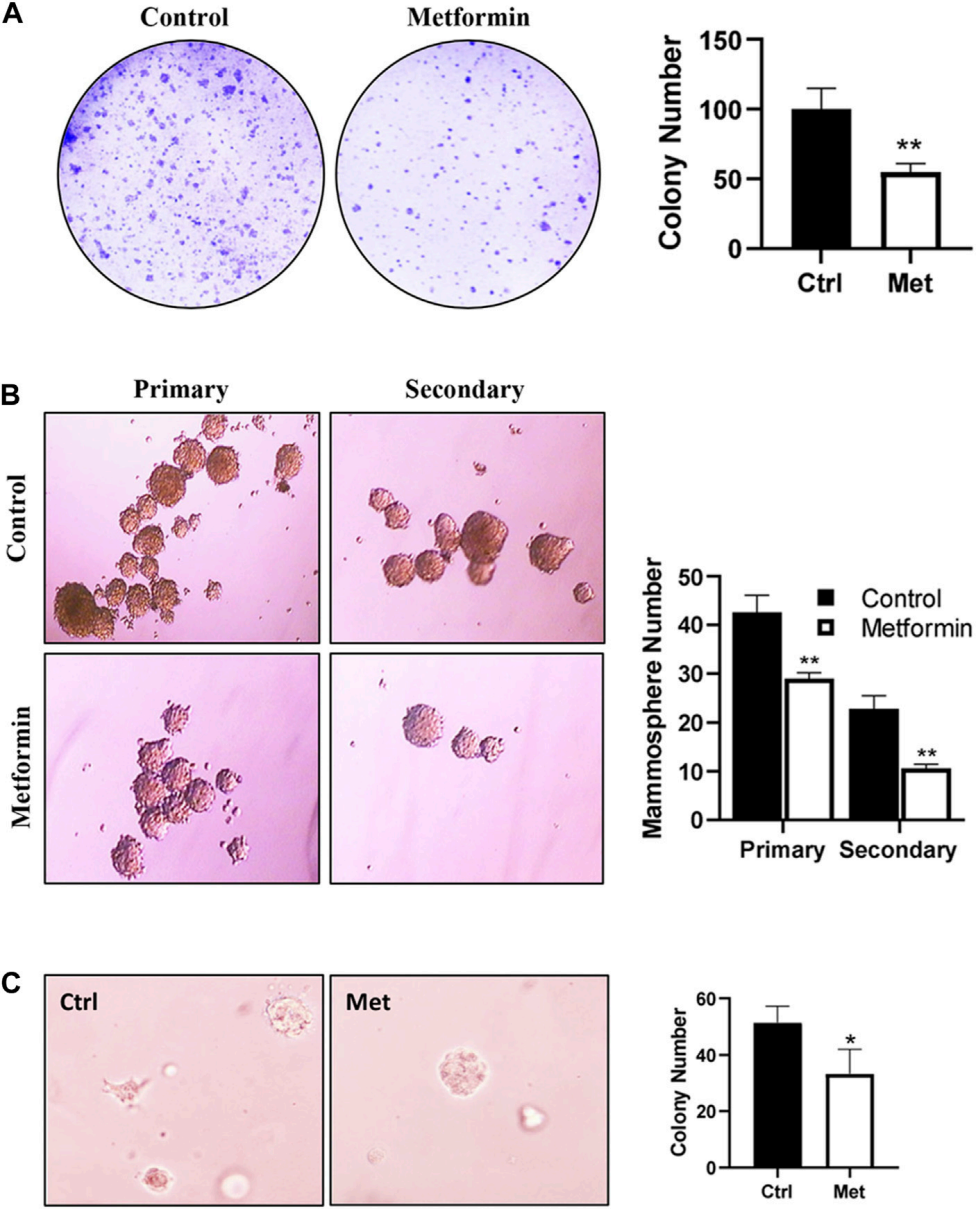
Metformin inhibits epithelial cell stemness of mammary tissues from FVB/N mice. Primary mammary epithelial cells (MECs) were isolated from control and metformin-treated mice (5 mice/group) and evaluated with functional assays for epithelial cell stemness. **(A)** Detection of relative numbers of mammary progenitor cells in the mammary tissues of control and metformin-treated mice with colony-forming cell (CFC) assays. CFC colonies formed in a dish inoculated with 4 × 10^3^ mammary cells from each group were documented. Results from triplicate experiments were quantified. **(B)** Mammosphere formation efficiency of MECs from control and metformin groups. Primary MECs were cultured in supplemented-Epicult-B media in triplicate for 7 days for the evaluation of primary mammospheres. Single cells from the primary spheres were cultured under the same conditions for secondary mammospheres. Spheres >30 μm were counted and quantified for statistical analysis. **(C)** Anchorage-independent growth of MECs was assessed with a 3D culture assay. Primary MECs from each group were grown in matrigel for 7 days, followed by fixation, staining, and colony analysis. Each of the assays was performed in triplicate (*p < 0.05, **p < 0.01).

To assess the effect of metformin on the functional potential of MaSCs and progenitor cells in FVB/N mice, we characterized MEC stemness using various functional assays. Colony-forming cell (CFC) assays were employed to detect the relative number of mammary progenitor cells in MECs (Stingl, 2009). Our results revealed a significant reduction in the colony formation efficiency of MECs in metformin-treated animals (Figure 3A), suggesting an inhibitory effect of metformin on MEC progenitor cells.

The mammosphere formation efficiency, especially that of secondary spheres, serves as an indicator of MaSC self-renewal potential (Shaw et al., 2012). Evaluation of MECs from control and metformin-treated FVB/N mice indicated a substantial reduction in mammosphere formation efficiency in the metformin group, particularly secondary mammosphere formation (Figure 3B). This suggests a significant attenuation of the self-renewal capabilities. Additionally, we assessed the effect of metformin on anchorage-independent growth of MECs using a 3D culture system. As depicted in Figure 3C, metformin-treated MECs exhibited a marked decrease in the number of 3D colonies formed in the semi-solid culture system, providing further evidence of metformin’s inhibitory effect of metformin on MEC stemness in FVB/N mice. Of note, the *in vitro* culture system for mammosphere, CFC assay and 3D culture was in the absence of metformin. Therefore, the phenotype changes between the control and metformin treated groups were attributed to *in vivo* metformin treatment.

### Metformin induces mammary epithelial cell reprogramming and stemness attenuation through regulation of AMPK/mTOR/S6K, PI3K/Akt and MAPK/Erk signaling pathways

**FIGURE 4:**
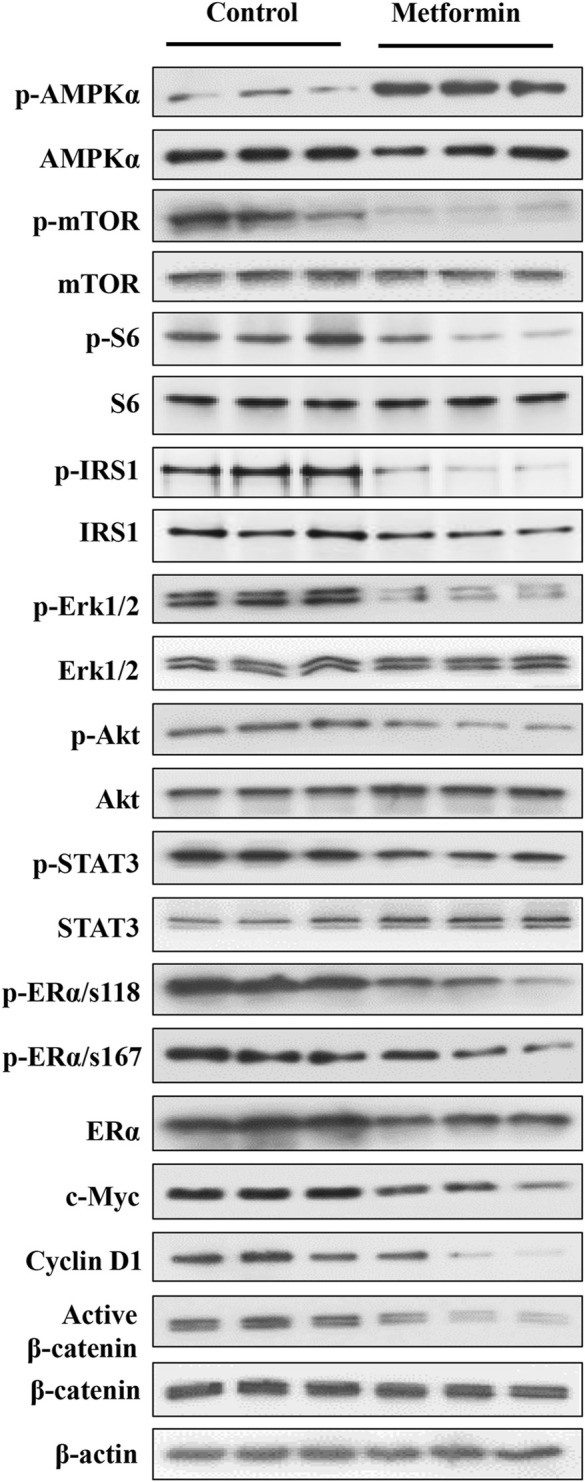
Western blot analysis of key markers in the AMPK, RTK, ER, and β-catenin pathways in mammary tissues of metformin-treated FVB/N mice. Protein lysates were prepared from mammary tissues of control and metformin-treated mice, followed by Western blotting. Protein levels of indicated markers from three mice of each group were analyzed. The markers encompass key regulators in AMPK, RTK, ER, and Wnt pathways to demonstrate metformin-modulated signaling in these pathways in the mammary tissues of FVB/N mice. The quantification of the relative protein levels and phosphorylation status of indicated markers is presented in Supplementary Figure S1.

Metformin-induced AMPK activation and the resultant inhibition of mTOR signaling is a major upstream cellular event (Howell et al., 2017). PI3K/Akt and MAPK/Erk signaling play a critical role in mammary development and tumorigenesis. Previously, we reported that metformin induces downregulation of signaling in the MAPK/Erk, PI3K/Akt/mTOR, and STAT3 pathways in mammary tissues of erbB2 transgenic mice, which have a background of elevated RTK signaling (Zhu et al., 2014; Zhao et al., 2020). Therefore, to elucidate the phenotypic alterations in FVB/N mice, we focused on the effect of metformin on the AMPK/mTOR, PI3K/Akt and MAPK/Erk pathways, reflecting the interaction between metformin and intrinsic regulatory signaling in mammary tissues. Our analysis of both the total and phosphorylated protein levels of key markers within these pathways revealed that metformin induced significant activation of AMPK and notable inhibition of mTOR and ribosomal protein S6 phosphorylation (Figure 4 and Supplementary Figure S1), supporting the role of AMPK/mTOR/S6K regulation in this process. Moreover, we specifically examined whether metformin-induced regulation of the mTORC1 pathway is due to a higher baseline activity of this pathway in mammary tissues. Our data indicate that signaling in this pathway was comparable between mammary and liver tissues (Supplementary Figure S2), suggesting that our observations are not limited to the mammary glands.

Importantly, our analysis also demonstrated a substantial reduction in the phosphorylation/activation of Erk1/2, Akt, and STAT3 in the metformin-treated group. These results underscore the role of the RTK pathway as a mediator of metformin-induced regulation in mammary tissues, as evidenced by the downstream effect of these markers on RTK signaling. We further demonstrated that phosphorylation of IRS1 (insulin receptor substrate 1) was significantly inhibited. Since it was reported that IRS1 is upstream of RTK/Akt/Erk signaling (Nie et al., 2016; Shi et al., 2021), our results support IRS1 as a mediator of metformin-induced inhibition of these pathways. The ER signaling pathway plays a crucial role in mammary development and breast cancer oncogenesis, acting as a central hub for various signaling interactions (Rusidzé et al., 2021). Previous investigations of mammary tissues from MMTV-erbB2 transgenic mice highlighted the intricate interplay between the ER and RTK pathways (Ma et al., 2017). In this study, metformin treatment induced a significant downregulation in both total ERα and phosphorylated ERα at S118, with a moderate decrease in p-ERα at S167 compared to the control (Figure 4). Additionally, in the context of changes in MEC stemness, our analysis of Wnt/β-catenin signaling revealed a downregulation of active β-catenin in the metformin-treated tissues. Concurrently, the protein levels of c-Myc and cyclin D1, downstream targets influenced by ER, Wnt/β-catenin, and other pathways, exhibited significant reductions. Together, our data provide compelling evidence that metformin induces AMPK signaling activation and downregulates the MAPK/Erk, PI3K/Akt, ER, and Wnt/β-catenin pathways. These findings suggest that regulation of these fundamental pathways plays a critical role in mediating metformin-induced MEC repopulation and stemness suppression in the mammary tissues of FVB/N mice.

### RNA sequencing analysis of gene expression profiles in mammary tissues induced by metformin treatment

**FIGURE 5:**
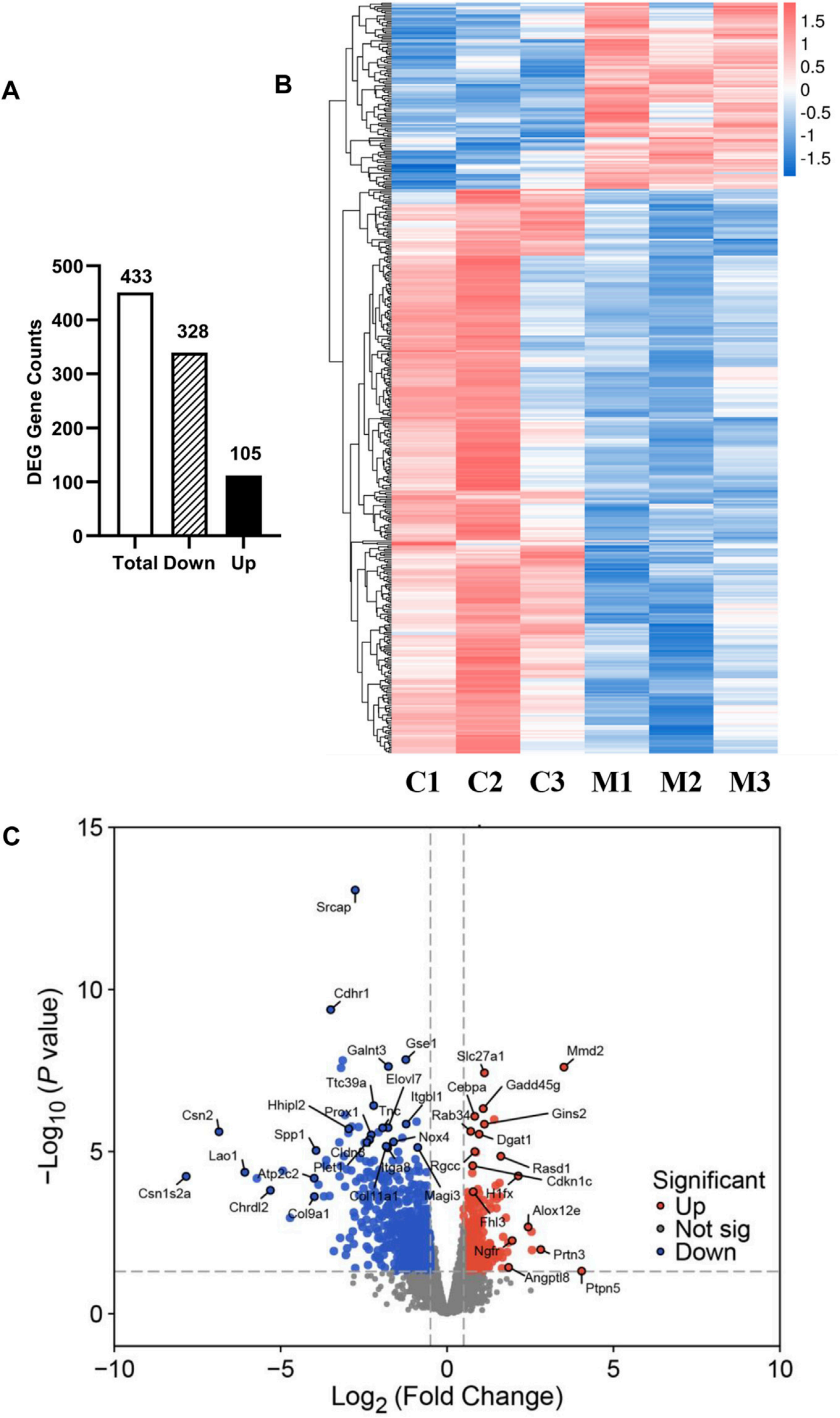
Overview of RNA sequencing analysis of gene expression profiles between control and metformin-treated FVB/N mice. **(A)** The numbers of differentially expressed genes (DEGs), including total, downregulated and upregulated genes, in the mammary tissues of control and metformin-treated mice. Criteria for DEGs were set as Fold Change (FC) ≥ 1.5 and *p*-value <0.01. **(B)** RNA-Seq heatmap showing the clusters of genes differentially expressed in mammary tissues of control (C1-3) and metformin-treated (M1-3) mice. **(C)** Volcano plot illustrating the 433 genes reaching statistical significance with downregulated genes in blue and upregulated genes in red.

To gain molecular insights into the mechanisms of metformin-induced regulation on MECs, we performed RNA-Seq analysis of gene expression profiles in mammary tissues of mice from each of the control and metformin groups. As summarized in Figure 5A, using a cutoff of >1.5-fold change and *p* < 0.01 for significance, we identified a total of 433 genes that were differentially expressed in the metformin group, as compared to the control. Notably, approximately three-quarters of these genes (328 genes) were downregulated, whereas 105 genes were upregulated, underscoring metformin’s inhibitory effect on gene expression associated with mammary proliferation and stemness. We next analyzed the DEGs using hierarchical clustering. The heat map depicted in Figure 5B revealed a predominant downregulation of gene expression among the differentially expressed genes. For specific annotation, the top 20 downregulated and top 15 upregulated genes among metformin-induced DEGs are summarized in Table 1. The functional context of these genes, and those on the extended list, are analyzed below. The volcano plot in Figure 5C not only displays the general gene expression profiles but also demonstrates top hits of DEGs based on statistical differences and fold changes. Among the top hits of the downregulated genes, *PLET1* is associated with mammary differentiation and stemness. Its downregulation could indicate reduced stemness or progenitor cell activity. *CHRDL2* is involved in BMP signaling and cell differentiation. *ITGA8*, *SPP1* and *COL9A1* genes are involved in cell adhesion, survival and extracellular matrix interactions. Downregulation of these genes suggests metformin inhibits cell migration, proliferation/differentiation, adhesion and glandular activity. Among the top hits of the upregulated genes, *H1FX* is the member X of histone family; *NGFR* (Nerve Growth Factor Receptor) and *PTPN5* are involved in cellular differentiation and cell cycle-related process. These changes appear to be relevant to the regulation of chromatin dynamics, cell proliferation/differentiation and possible stemness. Upregulation of *GADD45G* and *MMD2* may suggest the involvement of cellular stress and DNA damage. These changes provide clues for further studies of metformin-induced regulation in mammary tissues. Taken together, the regulatory pattern of the DEGs confirms the general inhibitory influence of metformin on mammary tissue gene expression and suggests a concerted suppression of specific pathways related to phenotypic changes. The complete list of DEGs is presented in Supplementary Table S1.

**TABLE 1:** Differentially expressed genes regulated by metformin.

Gene	Symbol	*p*-Value	log2FC	Regulated	Annotation
ENSMUSG00000061937	*CSN1S2A*	5.81E-05	−7.8400	down	alpha-S2-casein-like A isoform 1 precursor
ENSMUSG00000063157	*CSN2*	2.44E-06	−6.8535	down	beta-casein isoform a precursor
ENSMUSG00000024903	*LAO1*	4.34E-05	−6.0769	down	L-amino acid oxidase 1 precursor
ENSMUSG00000070702	*CSN1S1*	6.71E-05	−5.7157	down	alpha-S1-casein isoform a precursor
ENSMUSG00000030732	*CHRDL2*	1.56E-04	−5.3087	down	chordin-like protein 2 isoform 2 precursor
ENSMUSG00000022991	*LALBA*	3.90E-05	−4.9353	down	alpha-lactalbumin precursor
ENSMUSG00000026818	*CEL*	1.10E-03	−4.7162	down	bile salt-activated lipase precursor
ENSMUSG00000034112	*ATP2C2*	6.61E-05	−3.9932	down	calcium-transporting ATPase type 2C member 2
ENSMUSG00000026147	*COL9A1*	2.45E-04	−3.9879	down	Collagen alpha-1(IX) chain; Flags: Precursor
ENSMUSG00000037145	*LYPD8l*	3.64E-08	−3.9580	down	epithelial progenitor 1 precursor
ENSMUSG00000029304	*SPP1*	9.24E-06	−3.9376	down	osteopontin isoform 4 precursor
ENSMUSG00000001827	*FOLR1*	1.04E-04	−3.8652	down	folate receptor alpha precursor
ENSMUSG00000029188	*SLC34A2*	2.42E-04	−3.7003	down	sodium-dependent phosphate transport protein 2B
ENSMUSG00000001622	*CSN3*	2.67E-05	−3.6477	down	kappa-casein isoform a precursor
NewGene_2453	*NEWGENE_2453*	1.83E-05	−3.6297	down	immunoglobulin light chain variable region, partial
NewGene_2472	*NEWGENE_2472*	2.33E-04	−3.5340	down	immunoglobulin light chain variable region, partial
ENSMUSG00000021803	*CDHR1*	4.19E-10	−3.4968	down	cadherin-related family member 1 precursor
NewGene_4750	*NEWGENE_4750*	5.18E-06	−3.2377	down	mCG129376
NewGene_2444	*NEWGENE_2444*	5.81E-05	−3.2311	down	mCG141631, partial
NewGene_2447	*NEWGENE_2447*	4.59E-03	−3.2104	down	mCG141625, partial
ENSMUSG00000039533	*MMD2*	2.48E-08	3.5132	up	monocyte to macrophage differentiation factor 2
ENSMUSG00000086962	*GM12248*	2.96E-03	2.5378	up	fatty acid hydroxylase domain-containing protein 2
ENSMUSG00000018907	*ALOX12E*	2.10E-03	2.4357	up	polyunsaturated fatty acid (12S)/(13S)-lipoxygenase, isoform X1
ENSMUSG00000044927	*H1FX*	5.64E-05	2.1381	up	H1 histone family, member X
ENSMUSG00000000120	*NGFR*	5.57E-03	1.9554	up	tumor necrosis factor receptor superfamily member 16 precursor
ENSMUSG00000053687	*DPEP2*	6.41E-03	1.8398	up	dipeptidase 2 isoform X1
ENSMUSG00000072624	*GM5460*	1.10E-03	1.7682	up	uncharacterized protein LOC432838 isoform X2
ENSMUSG00000009394	*SYN2*	4.27E-03	1.6855	up	synapsin-2 isoform IIb
ENSMUSG00000049892	*RASD1*	1.39E-05	1.6156	up	dexamethasone-induced Ras-related protein 1 [Arvicanthis niloticus]
ENSMUSG00000059824	*DBP*	2.42E-04	1.5917	up	D site-binding protein
ENSMUSG00000071497	*NUTF2-PS1*	9.24E-05	1.5730	up	nuclear transport factor 2 isoform X2 [*Castor canadensis*]
ENSMUSG00000056487	*METTL7A2*	2.90E-04	1.4918	up	methyltransferase like 7A2
ENSMUSG00000010307	*TMEM86A*	5.53E-04	1.4862	up	lysoplasmalogenase-like protein TMEM86A
ENSMUSG00000020131	*PCSK4*	1.12E-04	1.4614	up	proprotein convertase subtilisin/kexin type 4 precursor
ENSMUSG00000046215	*RPRML*	1.02E-06	1.4154	up	reprimo-like protein
ENSMUSG00000095789	*NUPR1L*	2.43E-04	1.3957	up	nuclear protein 2
ENSMUSG00000095562	*GM21887*	9.93E-03	1.3521	up	erythroid differentiation regulator

### Gene ontology (GO) analysis of DEGs in mammary tissues of control and metformin-treated mice

**FIGURE 6:**
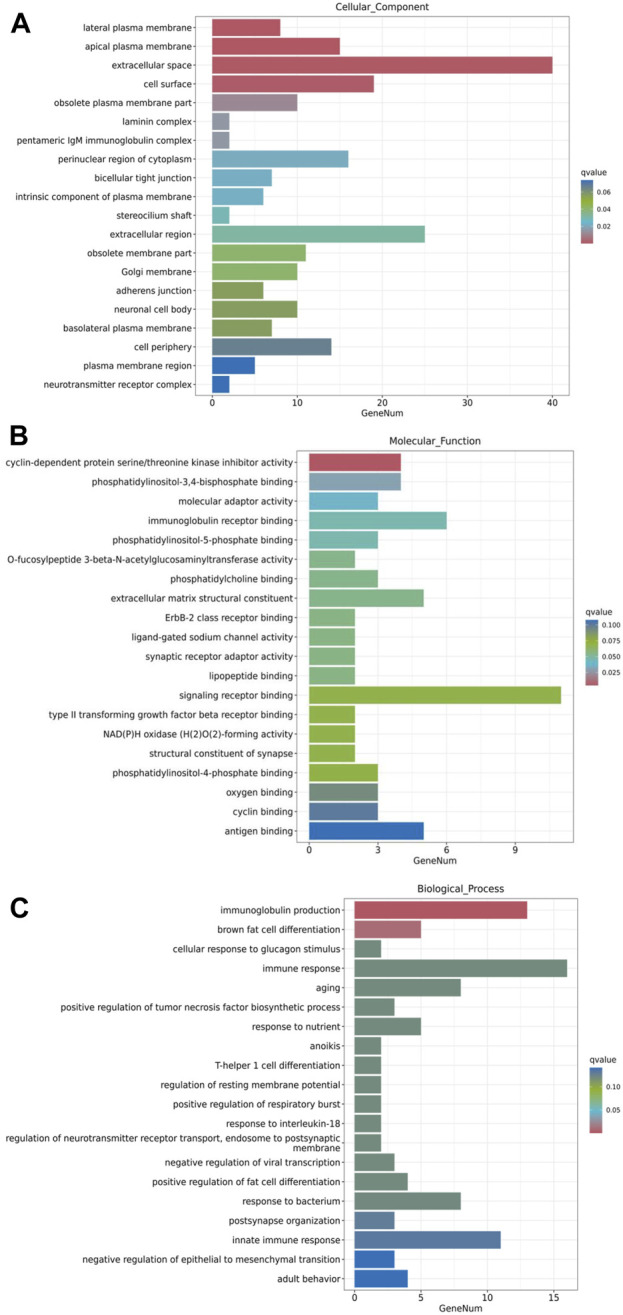
GO enrichment analysis of metformin-induced DEGs in mammary tissues of FVB/N mice. The top 20 gene sets based on cellular component, molecular function, and biological process were presented in **(A–C)**, respectively. The *x*-axis displays a number of differentially expressed genes (GeneNum); The *y*-axis indicates GO terms. The gene sets were listed in order based on q value (on the right) in the analysis.

GO enrichment analysis was performed to understand the functional context of the DEGs relative to cellular component, molecular function and biological process. The results revealed that, in terms of cellular component, the major groups of DEGs were associated with lateral plasma membrane, apical plasma membrane, extracellular space, cell surface and obsolete plasma membrane part (Figure 6A). In terms of molecular function, the major DEG groups were related to cyclin-dependent protein serine/threonine kinase inhibitor activity, phosphatidylinositol-3,4-bisphosphate binding, molecular adaptor activity, immunoglobulin receptor binding, and phosphatidylinositol-5-phosphate binding (Figure 6B). The major biological processes involving the DEGs were immunoglobulin production, brown fat cell differentiation, cellular response to glucagon stimulus, immune response and aging (Figure 6C). While comprehensive analysis of the full lists may reveal a more in-depth understanding, these prominent changes in GO analysis suggest that the DEGs associated with extracellular components, transmembrane signaling and regulation of immune responses are of significance for further investigation on metformin-induced gene expression.

### KEGG analysis of the DEGs for the pathways regulated by metformin

**FIGURE 7:**
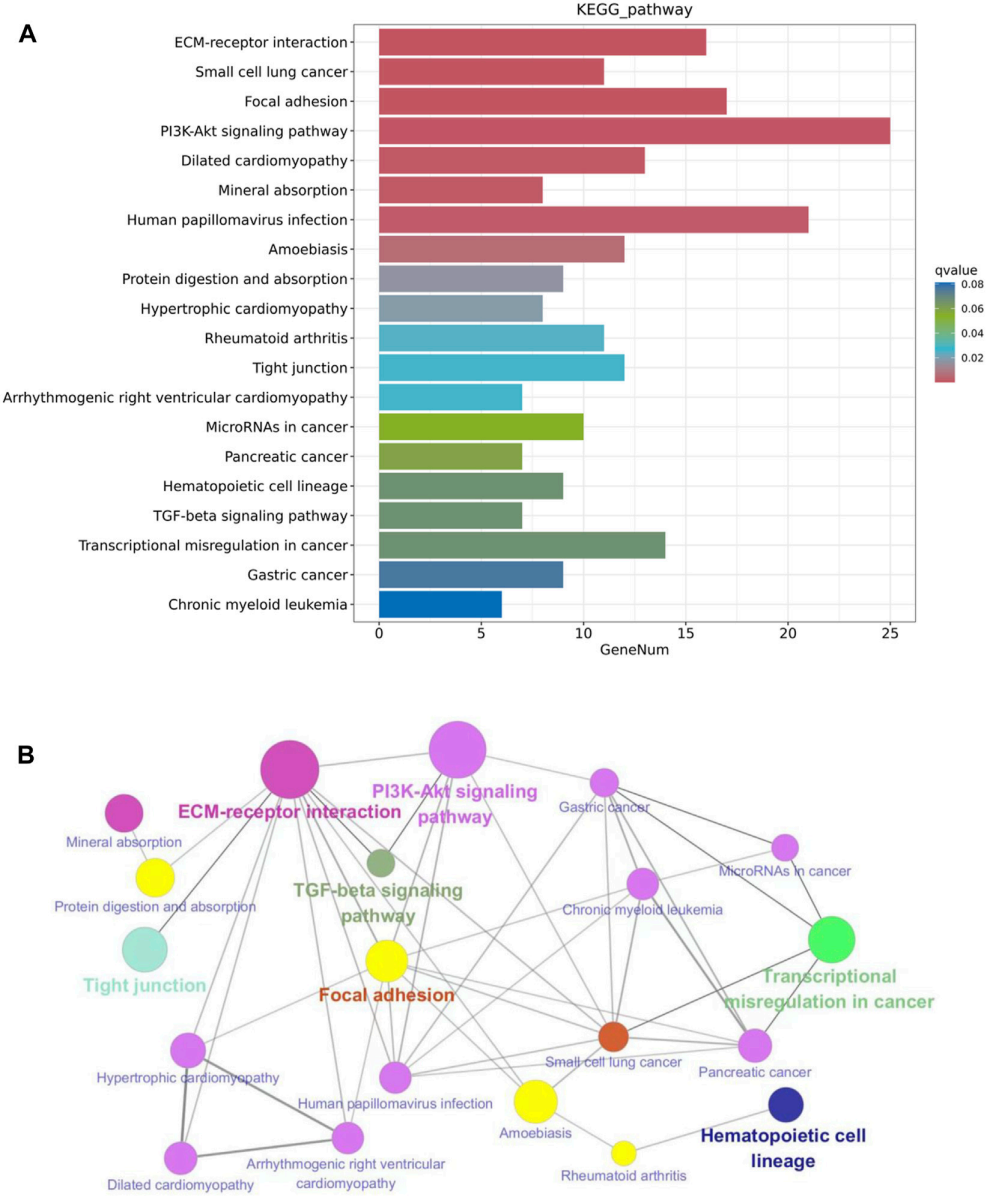
KEGG pathway enrichment analysis of metformin-induced DEGs in mammary tissues of FVB/N mice. **(A)** The top 20 KEGG pathways of the DEGs based on q value (on the right) are presented. *X*-axis displays a number of differentially expressed genes (GeneNum); The *y*-axis indicates specified pathways. Specific genes of individual pathways corresponding to each bar are listed in Table 2. **(B)** Graphical visualization of the network of key KEGG pathways induced by metformin treatment, with node sizes proportional to their corresponding Z-scores.

The KEGG pathway enrichment analysis reveals potential links between genetic information of the DEGs and cellular functions that are significantly modulated by specific treatments. As shown in Figure 7A, results from the KEGG analysis of metformin-induced DEGs indicate that the ECM-receptor interaction pathway was the most prominently enriched pathway. Pathways implicated in cancer, such as small cell lung cancer and microRNAs in cancer, exhibited considerable enrichment. Pathways involved in membrane receptor signaling, including the PI3K/Akt signaling pathway, TGF-β signaling pathway, focal adhesion, and tight junction pathways, were also notably regulated. Moreover, pathways relevant to metabolism, such as mineral absorption and cardiomyopathy-related genes were also among top regulated pathways. The specific genes involved in the top 20 KEGG pathways that were differentially regulated by metformin are summarized in Table 2. Results from these analyses suggest a potential influence of metformin treatment on critical pathways involved in oncogenic processes, metabolism, and fundamental cell signaling. To explore the potential connections among the top 20 KEGG pathways identified from enrichment analysis, we constructed the interaction network and visualization map using Cytoscape. As shown in Figure 7B, the major pathways in the network that interact with other pathways are the ECM-receptor interaction pathway, the PI3K-Akt signaling pathway, and the focal adhesion pathway. Integrating these pathways will enhance our understanding of the underlying mechanisms and provide guidance for further functional analysis. It is important to note that the data in Figure 7 focuses on the top 20 KEGG pathways. The data from the RNA-Seq project encompassed more complex and rich datasets. In addition to the aforementioned analysis, we also performed KEGG pathway enrichment analysis based on the total list of DEGs, which included both upregulated and downregulated genes, as well as upregulated and downregulated DEG lists respectively (see Supplementary Figure S3). This extended analysis provides additional insights for further studies.

**TABLE 2:** Enriched DEGs in KEGG pathways.

KEGG ID	KEGG pathways	Enriched DEGs	Gene Counts
ko04512	ECM-receptor interaction	*LAMA3; ITGB8; COL9A1; LAMB3; ITGA8; ITGA6; TNC; SPP1; COL4A5; LAMA1; ITGBL1; ITGA2B; FREM2; COL6A6; SV2C; COL6A5*	16
ko05222	Small cell lung cancer	*RXRG; GADD45G; RB1; CDKN1A; LAMA3; LAMB3; ITGA6; COL4A5; LAMA1; ITGA2B; CDKN2B*	11
ko04510	Focal adhesion	*LAMA3; VEGFB; ITGB8; COL9A1; RALGPS2; LAMB3; ITGA8; ITGA6; TNC; SPP1; COL4A5; LAMA1; ITGBL1; ITGA2B; NRN1; COL6A6; COL6A5*	17
ko04151	PI3K-Akt signaling pathway	*NGFR; ERBB3; HSP90AA1; CDKN1A; LAMA3; VEGFB; ITGB8; COL9A1; LAMB3; ITGA8; ITGA6; TNC; SPP1; COL4A5; LAMA1; ITGBL1; ITGA2B; TLR4; COL6A6; IL3RA; COL6A5; NEWGENE_4742; NEWGENE_4750; NEWGENE_4757; NEWGENE_5304*	25
ko05414	Dilated cardiomyopathy	*TGFB3; ITGB8; ITGA8; ITGA6; LAMA1; ITGBL1; ITGA2B; ADRB1; TGFB2; NEWGENE_4742; NEWGENE_4750; NEWGENE_4757; NEWGENE_5304*	13
ko04978	Mineral absorption	*SLC5A1; VDR; CLCN2; SLC40A1; SLC34A2; MT2; TRF; ATP1A1*	8
ko05165	Human papillomavirus infection	*ATP6V1B1; MFNG; RB1; CDKN1A; LAMA3; ITGB8; COL9A1; LAMB3; ITGA8; ITGA6; ATP6V0D2; TNC; SPP1; LFNG; COL4A5; LAMA1; ITGBL1; ITGA2B; COL6A6; PATJ; COL6A5*	21
ko05146	Amoebiasis	*TGFB3; LAMA3; LAMB3; CD1D1; COL4A5; LAMA1; TLR4; TGFB2; NEWGENE_4742; NEWGENE_4750; NEWGENE_4757; NEWGENE_5304*	12
ko04974	Protein digestion and absorption	*KCNK5; COL9A1; COL11A1; COL4A5; ATP1A1; COL6A6; KCNN4; COL8A1; COL6A5*	9
ko05410	Hypertrophic cardiomyopathy	*TGFB3; ITGB8; ITGA8; ITGA6; LAMA1; ITGBL1; ITGA2B; TGFB2*	8
ko05323	Rheumatoid arthritis	*ATP6V1B1; TGFB3; TNFRSF11A; ATP6V0D2; TLR4; TGFB2; GM49392; NEWGENE_4742; NEWGENE_4750; NEWGENE_4757; NEWGENE_5304*	11
ko04530	Tight junction	*IGSF5; MARVELD3; CLDN15; MYH11; OCLN; RAI14; CLDN1; CD1D1; EPB41L4B; CLDN8; PATJ; CGN*	12
ko05412	Arrhythmogenic right ventricular cardiomyopathy	*ITGB8; ITGA8; ITGA6; LAMA1; ITGBL1; ITGA2B; DSG2*	7
ko05206	MicroRNAs in cancer	*ERBB3; CDKN1A; SLC45A3; MMP16; TNC; ST14; IRS2; TGFB2; BMF; SERPINB5*	10
ko05212	Pancreatic cancer	*TGFBR1; TGFB3; GADD45G; RB1; CDKN1A; ARHGEF6; TGFB2*	7
ko04640	Hematopoietic cell lineage	*ITGA6; CD1D1; ITGA2B; CD24A; IL3RA; NEWGENE_4742; NEWGENE_4750; NEWGENE_4757; NEWGENE_5304*	9
ko04350	TGF-beta signaling pathway	*CHRD; TGFBR1; ID2; TGFB3; TGFB2; ID1; CDKN2B*	7
ko05202	Transcriptional misregulation in cancer	*NGFR; RXRG; ID2; GADD45G; CDKN1A; SLC45A3; SPINT1; CEBPA; LMO1; NUPR1L; NEWGENE_4742; NEWGENE_4750; NEWGENE_4757; NEWGENE_5304*	14
ko05226	Gastric cancer	*CDH1; TGFBR1; RXRG; TGFB3; GADD45G; RB1; CDKN1A; TGFB2; CDKN2B*	9
ko05220	Chronic myeloid leukemia	*TGFBR1; TGFB3; GADD45G; RB1; CDKN1A; TGFB2*	6

## Discussion

The findings of this study significantly contribute to our understanding of metformin-induced cellular responses in normal mammary tissues and their potential for breast cancer prevention. Using a non-tumor FVB/N mouse model, we identified alterations in mammary gland development, MEC repopulation, and signal transduction that align with the proposed antineoplastic mechanisms of metformin. These results not only support the hypothesis that metformin can modulate early cellular events predisposing to cancer cells but also underscore the importance of utilizing non-tumor models for mechanistic insights.

Our study shifted the conventional focus from treating established tumors to the critical window of cancer prevention. We aimed to demonstrate that metformin modulates specific intrinsic regulatory networks in premalignant tissues, reducing their susceptibility to genetic mutations and environmental carcinogens, thereby supporting the role of metformin in breast cancer prevention. The observed phenotypic changes, including reduced proliferation in normal mammary tissues, as evidenced by wholemount and Ki67 analyses (Figure 1), suggested that metformin exerts inhibitory effects that may prevent the unchecked cellular expansion characteristic of early cancer stages. The attenuation of epithelial cell proliferation by metformin may serve as a barrier to unchecked cellular expansion often observed during the early stages of cancer development (Pedraza-Fariña, 2006). These findings align with epidemiological data showing a lower cancer incidence in patients with diabetes treated with metformin (Col et al., 2012), highlighting its potential for chemoprevention.

In this study, metformin was administered at a dose of 200 mg/kg/day. Dose selection was based on previous studies on metformin-associated anti-cancer activities using various mouse models, which used metformin doses ranging from 100 to 250 mg/kg/day (Wu et al., 2012; Chen et al., 2017; Howell et al., 2017). Although this dose falls within the high-dose range for most models, it has been suggested that the effect of the standard dose of metformin used in humans for the treatment of type-2 diabetes (∼20 mg/kg) is equivalent to that of the ∼250 mg/kg dose used in mice, despite the considerably higher absolute dose in mice compared to humans (Howell et al., 2017; Meng et al., 2020). Therefore, the selected dose in our study was clinically relevant. The dose-response effect of metformin-induced mammary reprogramming should be examined in future studies to achieve maximal benefits at lower metformin doses.

Our results demonstrate that metformin significantly reduced the basal cell subpopulation and concurrently decreased both the MRU and CD61+/CD49f+ cell subpopulations (Figure 2). These findings suggest that metformin interacts with the intrinsic pathways to reprogram the hierarchy of MECs. Given that basal MECs are enriched with MaSCs, and the CD61+/CD49f+ subpopulation is enriched with progenitor cells (Shackleton et al., 2006), these alterations indicate that metformin inhibits the stemness of MECs/MaSCs. This conclusion is further supported by our stemness analysis data, including mammosphere, CFC, and 3D culture assays (Figure 3). Our findings underscore the effect of metformin on slowing MaSC self-renewal dynamics, potentially acting as a barrier to oncogenic factor-induced tumor initiation. Notably, our findings align with a study showing that metformin reduces the HR+ luminal subpopulation and their progenitors in normal C57BL/6 mice (Shehata et al., 2019). Although both studies suggest that metformin reprograms MECs, they differ in the targeted subpopulation (basal vs. luminal). The discrepancy in metformin-targeted subpopulations between the two systems, whether due to different mouse strains or different examination approaches, warrants further investigation. Future studies comparing both models under identical settings could address this issue. In the context of our previous study (Zhu et al., 2014), results from both MMTV-erbB2 transgenic mice and the parental non-tumor FVB/N strain suggested that the basal subpopulation is the primary cellular target of metformin in premalignant tissues, indicating that metformin-induced MEC reprogramming is not limited to tissues with oncogene overexpression. Data from these models suggest that the inhibition of proliferation and morphogenesis by metformin is linked to a reduction in MEC/MaSC stemness and the induction of MEC reprogramming. Given the critical role of MaSC regulation in modulating breast cancer risk, the effects of metformin-induced MEC reprogramming should be further explored. Moreover, as an increasing number of reports suggest that basal-like breast cancer (BLBC) may originate from the dysregulation of basal mammary epithelial cells prior to malignant transformation (Valentin et al., 2012), our finding that metformin selectively targets the basal subpopulation suggests that metformin may be useful in the prevention and treatment of BLBC in clinical settings. Although we observed that metformin-induced mammary epithelial cell reprogramming was mainly in the basal subpopulation in our model, it does not exclude the changes of other subpopulations, as suggested by other reports (Shehata et al., 2019). Furthermore, given the potential association of aberrant basal subpopulations and the development of BLBC (Gastaldi et al., 2013), targeting basal subpopulations could be a useful strategy for breast cancer prevention and therapy beyond metformin treatment. These questions warrant further investigation in future studies.

Our results in mechanistic studies demonstrated that metformin treatment induced significant downregulation of signaling in the AMPK/mTOR/S6K, the IRS1/Akt/Erk-ER crosstalk and Wnt/β-catenin activities in mammary tissues (Figure 4). These changes provided fundamental support for the magnetic connections between drug treatment and the altered phenotypes. Significant inhibition of S6 phosphorylation/activation underscores the regulation of mTORC1 in metformin-induced inhibition of mammary stemness and suggests potential significance of targeting mTORC1 in breast cancer prevention. Indeed, our recent data showed that rapamycin treatment significantly inhibited syngeneic tumor growth and mammary stem cells in MMTV-erbB2 transgenic mice (unpublished data). These findings support further investigation on mTORC1 targeting in breast cancer prevention. Moreover, metformin-induced AMPK activation highlights its regulation on cellular metabolism in this process. AMPK can be activated by inhibition of the respiratory complex I in mitochondria (Herzig and Shaw, 2018). Interestingly, recent reports have shown that metformin reduces the rate of oxidative respiration in mammary epithelial cells (Shehata et al., 2019). In light of this, the observed reprogramming of MEC subpopulations and stemness in this study appears to be connected to metformin’s inhibition of mitochondrial respiration and its associated metabolic regulation. Future studies will delve deeper into this connection by analyzing mitochondrial function and metabolism within specific subpopulations.

Since the Akt/Erk-ER crosstalk and Wnt/β-catenin-mediated regulation play critical roles in mammary development and carcinogenesis. (Li et al., 2022), our evidence demonstrates that metformin-induced inhibition of IRS1, PI3K/Akt, MAPK/Erk, Wnt/β-catenin pathways, and ER signaling (Figure 4) bridges the gap between metformin-induced modulation of the AMPK/mTOR pathway and the regulation of MEC proliferation and reprogramming. Specifically, the PI3K/Akt pathway regulates proliferation, differentiation, and apoptosis of mammary epithelial cells. Mutations in components of this pathway are found in around 70% of breast cancers, which are also attractive therapeutic targets (Wickenden and Watson, 2010). MAPK/Erk is a critical regulator that mediates the signaling between RTKs and amplified cellular activities that are involved in mammary gland development and breast cancer progression (Whyte et al., 2009). In particular, phosphorylation/activation of ERα by Akt and/or Erk is essential for the RTK-ER crosstalk (Shou et al., 2004). Moreover, the Wnt/β-catenin pathway plays an instrumental role in regulating MaSCs and mammary development (Yu et al., 2016). Aberrant signaling in this pathway contributes to tumor initiation and development through cancer stem cell promotion (Reya and Clevers, 2005). Changes in these pathways in metformin-treated mammary tissues of the FVB/N model shed light on the intricate interplay between metformin and signaling pathways in mammary tissues that leads to MEC reprogramming. We previously reported that metformin induces similar effects on these pathways in the MMTV-erbB2 transgenic mouse model (Zhao et al., 2020). However, because these transgenic mice have overexpression of erbB-2 and elevated RTK-ER signaling activities, the effect of metformin on these pathways at background levels was not clear. The data in this report underscore the interaction between metformin and these pathways in the intrinsic signaling networks and their association with phenotypic changes. These findings suggest that the metformin-mediated inhibition of MEC proliferation in premalignant tissues is not limited to individuals predisposed to RTK superactivation. Notably, our cell signaling results provide initial evidence for the involvement of the key pathways discussed above, suggesting a proof of concept. Further studies are required to elucidate the detailed molecular mechanisms underlying this process. In particular, our observation that metformin induces simultaneous inhibition of MaSCs and Wnt signaling suggests that the downregulation of the Wnt/β-catenin pathway and its interaction with the RTK and ER pathways could play a crucial role in this context. Future identification of immediate targets within the Wnt pathway and employing innovative approaches such as transcriptome analysis will yield deeper insights into the mechanisms by which metformin induces the reprogramming of mammary gland stemness in MEC subpopulations.

Our transcriptomic analysis revealed significant changes in the gene expression profiles of mammary tissues treated with metformin compared to controls. We observed a majority of differentially expressed genes (DEGs) were downregulated (328 genes vs. 105 upregulated), indicating a general inhibitory effect of metformin on gene expression that might impact MEC proliferation and stemness. This aligns with our goal to understand how metformin prevents mammary gland development. The data generated from this experiment, including gene expression profiles and candidate pathways, has provided substantial insights. Gene clustering and heatmap analysis have highlighted these inhibitory gene expression profiles, potentially explaining the reduced proliferation rate of MECs. Additionally, volcano plot analysis, which considers both fold changes and statistical significances, offered a list of top hits for further testing of individual genes that contribute to altered phenotypes. Pathway enrichment analysis has been particularly revealing, identifying several pathways pertinent to metformin-induced reprogramming of mammary cells. Key pathways among these are the PI3K/Akt and TGF-β pathways, crucial for mammary gland development and carcinogenesis (Maharati and Moghbeli, 2023), which were consistently noted in our signal transduction results. Emerging pathways like ECM-receptor interaction and focal adhesion, crucial for regulating tissue plasticity and stemness, are highlighted as novel areas that might play a significant role in the reprogramming process. Furthermore, functional enrichment and GO analyses pinpointed the modulation of cellular communication and signaling, with enriched categories related to cell surface and extracellular components. Immune-related terms, such as immunoglobulin production and immune response, suggest potential immunomodulatory effects of metformin. KEGG pathway analysis indicated significant enrichment in cancer-related pathways, including small cell lung cancer and microRNAs in cancer, suggesting that metformin might interfere with key oncogenic pathways. Overall, our RNA-Seq analysis provides compelling evidence that metformin induces distinctive transcriptomic changes in mammary tissues, targeting pathways relevant to its preventive effects against mammary gland development. The identified genes and pathways warrant further investigation to elucidate the precise mechanisms of metformin-mediated prevention.

Furthermore, the variability in response to metformin treatment observed among individuals highlights the need for personalized approaches to breast cancer prevention. As we move towards precision medicine, understanding the genetic, metabolic, and environmental factors that influence metformin’s efficacy will be crucial for the development of tailored preventive strategies. Moreover, the application of novel technologies, such as single-cell sequencing and digital spatial profiling, may provide in-depth insight into metformin-induced differential regulation of MEC subpopulations and mammary stemness.

In conclusion, our results demonstrate that metformin induces the inhibition of MEC proliferation and morphogenesis, with basal and MRU subpopulations identified as major cellular targets of metformin in normal mammary tissues. These phenotypic changes are associated with the downregulation of signaling in the RTK and ER pathways, resulting from the interaction of metformin with intrinsic pathways. Our study underscores the utility of non-tumor models for determining the effects of metformin on MEC repopulation and stemness in premalignant mammary tissues and provides a framework for future research on the preventive applications of metformin in breast cancer.

## Data Availability

The datasets generated for this study are available in the Gene Expression Omnibus (GEO) repository at the National Center for Biotechnology Information (NCBI) under accession number GSE269308.
