# Supplementary material for: Metformin reduces basal subpopulation and attenuates mammary epithelial cell stemness in FVB/N mice
Source: Front Cell Dev Biol. 2024 Jul 11;12:1427395. doi: 10.3389/fcell.2024.1427395 (PMC11269140; doi:10.3389/fcell.2024.1427395)
Supplement: Supplementary file 1 [file Table1.DOCX]

| **Supplementary Table 1: Differentially Expressed Genes Between Metformin treated and Control Groups** | | | | | |
| --- | --- | --- | --- | --- | --- |
|  |  |  |  |  |  |
| **Gene** | **Symbol** | **P value** | **log2FC** | **Regulated** | **Annotation** |
| ENSMUSG00000032796 | *LAMA1* | 2.20E-03 | -1.2985 | down | laminin subunit alpha-1 precursor |
| ENSMUSG00000042784 | *MUC1* | 6.10E-04 | -2.1273 | down | mucin-1 precursor |
| ENSMUSG00000070407 | *HS3ST3B1* | 6.42E-03 | -0.7017 | down | heparan sulfate glucosamine 3-O-sulfotransferase 3B1 |
| ENSMUSG00000043719 | *COL6A6* | 2.17E-03 | -1.5639 | down | collagen alpha-6(VI) chain isoform 1 precursor |
| ENSMUSG00000023243 | *KCNK5* | 2.29E-03 | -1.4103 | down | potassium channel subfamily K member 5 |
| ENSMUSG00000028238 | *ATP6V0D2* | 2.84E-03 | -2.0764 | down | V-type proton ATPase subunit d 2 |
| ENSMUSG00000001763 | *TSPAN33* | 7.19E-03 | -1.6809 | down | tetraspanin-33 isoform 1 |
| ENSMUSG00000026147 | *COL9A1* | 2.45E-04 | -3.9879 | down | RecName: Full=Collagen alpha-1(IX) chain; Flags: Precursor |
| ENSMUSG00000030873 | *SCNN1B* | 5.32E-06 | -2.4728 | down | unnamed protein product |
| ENSMUSG00000031822 | *GSE1* | 1.46E-08 | -1.2404 | down | genetic suppressor element 1 isoform 1 |
| ENSMUSG00000058248 | *KCNH1* | 8.00E-04 | -1.6529 | down | potassium voltage-gated channel subfamily H member 1 isoform 1 |
| ENSMUSG00000029234 | *TMEM165* | 4.17E-03 | -0.7704 | down | transmembrane protein 165 precursor |
| ENSMUSG00000031647 | *MFAP3L* | 7.78E-04 | -0.7682 | down | microfibrillar-associated protein 3-like isoform a precursor |
| ENSMUSG00000039005 | *TLR4* | 3.39E-03 | -0.7267 | down | toll-like receptor 4 precursor |
| ENSMUSG00000026158 | *OGFRL1* | 8.28E-04 | -1.8252 | down | opioid growth factor receptor-like protein 1 isoform 2 |
| ENSMUSG00000022780 | *MELTF* | 1.82E-03 | -1.7827 | down | melanotransferrin precursor |
| ENSMUSG00000022105 | *RB1* | 2.47E-04 | -0.7427 | down | retinoblastoma-associated protein |
| ENSMUSG00000037541 | *SHANK2* | 4.29E-05 | -2.4043 | down | SH3 and multiple ankyrin repeat domains protein 2 isoform X13 |
| ENSMUSG00000079620 | *MUC4* | 2.51E-03 | -2.3254 | down | mucin family of glycoproteins |
| NewGene_2474 | *NEWGENE_2474* | 4.16E-05 | -2.3534 | down | mCG1036441, partial |
| ENSMUSG00000040969 | *ARHGEF38* | 2.66E-06 | -2.9653 | down | rho guanine nucleotide exchange factor 38 isoform 1 |
| ENSMUSG00000043782 | *BICDL2* | 3.38E-03 | -1.5978 | down | BICD family-like cargo adapter 2 |
| ENSMUSG00000022286 | *GRHL2* | 3.31E-03 | -1.2455 | down | grainyhead-like 2 (Drosophila), isoform CRA_a, partial |
| ENSMUSG00000019779 | *FRK* | 4.92E-03 | -1.1105 | down | fyn-related kinase, isoform CRA_b, partial |
| ENSMUSG00000001622 | *CSN3* | 2.67E-05 | -3.6477 | down | kappa-casein isoform a precursor |
| NewGene_4757 | *NEWGENE_4757* | 1.39E-05 | -3.0971 | down | mCG129879, partial |
| ENSMUSG00000058145 | *ADAMTS17* | 4.85E-04 | -2.3905 | down | A disintegrin and metalloproteinase with thrombospondin motifs 17 precursor |
| ENSMUSG00000029188 | *SLC34A2* | 2.42E-04 | -3.7003 | down | sodium-dependent phosphate transport protein 2B |
| ENSMUSG00000000159 | *IGSF5* | 1.73E-04 | -2.1693 | down | immunoglobulin superfamily member 5 isoform 2 precursor |
| ENSMUSG00000074715 | *CCL28* | 3.19E-04 | -2.5102 | down | C-C motif chemokine 28 precursor |
| ENSMUSG00000060512 | *0610040J01RIK* | 9.51E-04 | -1.4657 | down | uncharacterized protein C4orf19 homolog |
| ENSMUSG00000035125 | *GCFC2* | 6.16E-03 | -0.8698 | down | GC-rich sequence DNA-binding factor 2 |
| ENSMUSG00000004885 | *CRABP2* | 1.72E-03 | -1.6143 | down | cellular retinoic acid-binding protein 2 |
| ENSMUSG00000076441 | *ASS1* | 6.10E-03 | -1.0827 | down | argininosuccinate synthase |
| ENSMUSG00000026638 | *IRF6* | 3.35E-04 | -1.1727 | down | interferon regulatory factor 6 |
| NewGene_4832 | *NEWGENE_4832* | 7.11E-03 | -1.3768 | down | predicted gene 7592 isoform X4 |
| ENSMUSG00000028555 | *TTC39A* | 3.81E-07 | -2.2051 | down | RIKEN cDNA 4922503N01, isoform CRA_d, partial |
| ENSMUSG00000026639 | *LAMB3* | 5.34E-04 | -1.8186 | down | laminin subunit beta-3 precursor |
| ENSMUSG00000030759 | *FAR1* | 7.99E-04 | -1.0809 | down | fatty acyl-CoA reductase 1 isoform X1 |
| ENSMUSG00000058729 | *LIN9* | 2.02E-03 | -0.6989 | down | protein lin-9 homolog |
| NewGene_2420 | *NEWGENE_2420* | 4.53E-03 | -2.0681 | down | mammary tumour virus 1, superantigen |
| NewGene_5129 | *NEWGENE_5129* | 1.20E-03 | -2.0435 | down | uncharacterized protein C030002A05Rik |
| ENSMUSG00000030729 | *PGM2L1* | 5.57E-03 | -0.6187 | down | glucose 1,6-bisphosphate synthase |
| ENSMUSG00000067149 | *JCHAIN* | 7.09E-03 | -2.8051 | down | immunoglobulin J chain precursor |
| ENSMUSG00000039239 | *TGFB2* | 1.61E-03 | -1.4578 | down | transforming growth factor beta-2 proprotein isoform 1 preproprotein |
| ENSMUSG00000051329 | *NUP160* | 6.10E-03 | -0.5943 | down | nuclear pore complex protein Nup160 |
| ENSMUSG00000005148 | *KLF5* | 2.28E-03 | -0.8593 | down | Kruppel-like factor 5, isoform CRA_a, partial |
| ENSMUSG00000044770 | *SCML4* | 3.30E-03 | -1.6936 | down | sex comb on midleg-like protein 4 isoform X1 |
| ENSMUSG00000030792 | *DKKL1* | 2.06E-04 | -1.6757 | down | dickkopf-like protein 1 precursor |
| ENSMUSG00000025813 | *HOMER2* | 1.51E-03 | -1.0097 | down | homer homolog 2 (Drosophila), isoform CRA_a, partial |
| ENSMUSG00000026594 | *RALGPS2* | 8.93E-03 | -0.8362 | down | ras-specific guanine nucleotide-releasing factor RalGPS2 isoform c |
| ENSMUSG00000027799 | *NBEA* | 9.53E-03 | -0.6181 | down | neurobeachin |
| ENSMUSG00000016024 | *LBP* | 8.48E-03 | -2.4120 | down | lipopolysaccharide-binding protein precursor |
| ENSMUSG00000032570 | *ATP2C1* | 4.30E-05 | -0.6072 | down | calcium-transporting ATPase type 2C member 1 isoform 2 |
| NewGene_2453 | *NEWGENE_2453* | 1.83E-05 | -3.6297 | down | immunoglobulin light chain variable region, partial |
| ENSMUSG00000032776 | *MCTP2* | 6.68E-03 | -1.0591 | down | multiple C2 and transmembrane domain-containing protein 2 |
| ENSMUSG00000026822 | *LCN2* | 2.29E-04 | -2.0660 | down | neutrophil gelatinase-associated lipocalin precursor |
| ENSMUSG00000044708 | *KCNJ10* | 2.99E-04 | -2.0290 | down | ATP-sensitive inward rectifier potassium channel 10 |
| ENSMUSG00000034607 | *POF1B* | 8.18E-04 | -1.4067 | down | protein POF1B |
| ENSMUSG00000033389 | *ARHGAP44* | 4.08E-03 | -0.7554 | down | rho GTPase-activating protein 44 isoform 2 |
| ENSMUSG00000028159 | *DAPP1* | 7.15E-03 | -0.8594 | down | adapter for phosphotyrosine and 3-phosphotyrosine/3-phosphoinositide |
| ENSMUSG00000025993 | *SLC40A1* | 8.65E-04 | -1.4091 | down | solute carrier family 40 member 1 |
| ENSMUSG00000042473 | *TBC1D8B* | 3.98E-05 | -0.9397 | down | TBC1 domain family member 8B |
| ENSMUSG00000016763 | *SCUBE1* | 8.48E-03 | -1.0530 | down | CUB and EGF-like domain-containing protein 1 isoform c precursor |
| ENSMUSG00000031274 | *COL4A5* | 1.70E-03 | -1.0982 | down | collagen alpha-5(IV) chain isoform X1 |
| ENSMUSG00000045094 | *ARHGEF37* | 4.90E-03 | -1.1683 | down | rho guanine nucleotide exchange factor 37 |
| ENSMUSG00000033998 | *KCNK1* | 5.77E-05 | -1.5083 | down | potassium channel subfamily K member 1 |
| ENSMUSG00000003309 | *AP1M2* | 2.84E-03 | -1.4564 | down | AP-1 complex subunit mu-2 isoform 1 |
| ENSMUSG00000028262 | *CLCA3A2* | 8.27E-05 | -1.7650 | down | chloride channel calcium activated 3A2 precursor |
| ENSMUSG00000020422 | *TNS3* | 5.48E-03 | -0.8779 | down | tensin-3 isoform X2 |
| ENSMUSG00000046807 | *LRRC75B* | 8.36E-03 | -1.2884 | down | expressed sequence AI646023, isoform CRA_a, partial |
| ENSMUSG00000052920 | *PRKG1* | 1.38E-03 | -1.0992 | down | cGMP-dependent protein kinase 1 isoform alpha |
| ENSMUSG00000030708 | *DNAJB13* | 3.01E-04 | -1.6908 | down | dnaJ homolog subfamily B member 13 |
| ENSMUSG00000028434 | *EPB41L4B* | 2.42E-03 | -0.9462 | down | band 4.1-like protein 4B isoform X2 |
| ENSMUSG00000035638 | *MUC20* | 4.20E-03 | -1.9221 | down | mucin-20 isoform a precursor |
| ENSMUSG00000031133 | *ARHGEF6* | 1.72E-05 | -0.9499 | down | rho guanine nucleotide exchange factor 6 isoform 1 |
| ENSMUSG00000030691 | *FCHSD2* | 5.95E-04 | -0.7075 | down | mKIAA0769 protein, partial |
| ENSMUSG00000050730 | *ARHGAP42* | 2.85E-03 | -0.8109 | down | rho GTPase-activating protein 42 isoform 3 |
| ENSMUSG00000021270 | *HSP90AA1* | 1.14E-04 | -0.7029 | down | unnamed protein product |
| ENSMUSG00000038894 | *IRS2* | 2.65E-03 | -0.7589 | down | insulin receptor substrate 2 |
| ENSMUSG00000024479 | *MAL2* | 1.05E-04 | -0.9745 | down | protein MAL2 |
| ENSMUSG00000021253 | *TGFB3* | 4.44E-03 | -1.2252 | down | transforming growth factor beta-3 proprotein precursor |
| ENSMUSG00000031438 | *RNF128* | 1.46E-03 | -0.9712 | down | ring finger protein 128, partial |
| ENSMUSG00000018830 | *MYH11* | 2.82E-05 | -0.8679 | down | myosin-11 isoform 2 |
| ENSMUSG00000037129 | *TMPRSS13* | 5.31E-05 | -2.1254 | down | transmembrane protease serine 13 |
| ENSMUSG00000027298 | *TYRO3* | 2.29E-03 | -1.5878 | down | tyrosine-protein kinase receptor TYRO3 isoform B |
| ENSMUSG00000052539 | *MAGI3* | 7.45E-06 | -0.8819 | down | membrane associated guanylate kinase |
| ENSMUSG00000034112 | *ATP2C2* | 6.61E-05 | -3.9932 | down | calcium-transporting ATPase type 2C member 2 |
| ENSMUSG00000005107 | *SLC2A9* | 6.80E-05 | -2.4856 | down | solute carrier family 2, facilitated glucose transporter member 9 isoform 1 |
| ENSMUSG00000030862 | *CPXM2* | 1.19E-03 | -0.9796 | down | inactive carboxypeptidase-like protein X2 precursor |
| ENSMUSG00000032925 | *ITGBL1* | 1.41E-06 | -1.2304 | down | integrin beta-like protein 1 precursor |
| ENSMUSG00000025757 | *HSPA4L* | 2.08E-03 | -0.7229 | down | heat shock protein 4 like, isoform CRA_c, partial |
| ENSMUSG00000031292 | *CDKL5* | 2.04E-04 | -0.9149 | down | cyclin-dependent kinase-like 5 [Mus caroli] |
| ENSMUSG00000030494 | *RHPN2* | 2.46E-03 | -1.1406 | down | rhophilin, Rho GTPase binding protein 2, isoform CRA_b, partial |
| ENSMUSG00000020638 | *CMPK2* | 3.33E-03 | -0.9005 | down | UMP-CMP kinase 2, mitochondrial precursor |
| ENSMUSG00000015619 | *GATA3* | 5.75E-03 | -1.1091 | down | trans-acting T-cell-specific transcription factor GATA-3 isoform 1 |
| ENSMUSG00000026185 | *IGFBP5* | 1.02E-04 | -1.0485 | down | insulin-like growth factor binding protein 5, isoform CRA_b, partial |
| ENSMUSG00000044976 | *WDR72* | 2.62E-04 | -2.3654 | down | WD repeat-containing protein 72 |
| ENSMUSG00000058656 | *SAMD12* | 7.03E-03 | -0.7086 | down | sterile alpha motif domain-containing protein 12 |
| ENSMUSG00000028364 | *TNC* | 1.85E-06 | -1.9344 | down | tenascin isoform 1 precursor |
| ENSMUSG00000022674 | *UBE2V2* | 1.19E-06 | -0.9161 | down | ubiquitin-conjugating enzyme E2 variant 2 isoform 1 |
| ENSMUSG00000038679 | *TRPS1* | 1.43E-04 | -1.3345 | down | zinc finger transcription factor Trps1 isoform 1 |
| ENSMUSG00000045790 | *CCDC149* | 3.45E-03 | -0.9421 | down | coiled-coil domain-containing protein 149 isoform 1 |
| ENSMUSG00000050520 | *CLDN8* | 4.30E-06 | -2.3290 | down | claudin-8 |
| ENSMUSG00000045180 | *SHROOM2* | 9.71E-03 | -0.6696 | down | protein Shroom2 isoform 1 |
| ENSMUSG00000047139 | *CD24A* | 1.04E-03 | -0.6766 | down | signal transducer CD24 precursor |
| ENSMUSG00000028211 | *TRP53INP1* | 1.83E-04 | -0.8971 | down | tumor protein p53-inducible nuclear protein 1 isoform 1 |
| ENSMUSG00000022629 | *KIF21A* | 1.68E-03 | -0.6988 | down | kinesin-like protein KIF21A isoform 1 |
| ENSMUSG00000076937 | *IGLC2* | 2.11E-03 | -2.5375 | down | Ig lambda-2 chain |
| ENSMUSG00000006269 | *ATP6V1B1* | 8.54E-05 | -1.8973 | down | V-type proton ATPase subunit B, kidney isoform |
| ENSMUSG00000020142 | *SLC1A4* | 4.88E-04 | -1.6481 | down | neutral amino acid transporter A |
| NewGene_2421 | *NEWGENE_2421* | 1.55E-03 | -2.5747 | down | Ig kappa V-region 24B, partial |
| ENSMUSG00000044626 | *LIPH* | 1.13E-04 | -2.2404 | down | lipase member H isoform 1 precursor |
| NewGene_799 | *NEWGENE_799* | 1.49E-03 | -2.2112 | down | uncharacterized protein LOC110307580 [Mus caroli] |
| ENSMUSG00000022548 | *APOD* | 1.61E-04 | -1.1411 | down | apolipoprotein D precursor |
| ENSMUSG00000015134 | *ALDH1A3* | 1.35E-03 | -1.1927 | down | aldehyde dehydrogenase family 1 member A3 |
| ENSMUSG00000022246 | *RAI14* | 1.12E-04 | -0.6094 | down | ankycorbin isoform 1 |
| ENSMUSG00000040209 | *ZFP704* | 1.50E-03 | -0.7885 | down | zinc finger protein 704 |
| ENSMUSG00000021830 | *TXNDC16* | 6.35E-03 | -0.6034 | down | thioredoxin domain-containing protein 16 isoform 1 precursor |
| ENSMUSG00000028403 | *ZDHHC21* | 2.21E-03 | -0.6089 | down | zinc finger, DHHC domain containing 21 |
| ENSMUSG00000032496 | *LTF* | 1.60E-05 | -2.6180 | down | lactotransferrin precursor |
| ENSMUSG00000041261 | *CAR8* | 9.70E-04 | -0.9093 | down | carbonic anhydrase-related protein |
| ENSMUSG00000068876 | *CGN* | 7.00E-04 | -1.2606 | down | cingulin isoform X1 |
| ENSMUSG00000049502 | *DTX3L* | 7.85E-03 | -0.6118 | down | E3 ubiquitin-protein ligase DTX3L |
| ENSMUSG00000003617 | *CP* | 2.89E-04 | -1.1858 | down | ceruloplasmin isoform X1 |
| ENSMUSG00000043487 | *ACOT6* | 6.86E-03 | -0.9480 | down | Acyl-CoA thioesterase 6 |
| ENSMUSG00000021365 | *NEDD9* | 1.30E-03 | -0.8061 | down | neural precursor cell expressed, developmentally down-regulated gene 9 |
| ENSMUSG00000067889 | *SPTBN2* | 4.80E-03 | -1.0883 | down | spectrin beta chain, non-erythrocytic 2 |
| ENSMUSG00000042766 | *TRIM46* | 9.05E-03 | -1.6845 | down | tripartite motif-containing protein 46 isoform X1 |
| ENSMUSG00000032718 | *MANSC1* | 3.09E-03 | -1.1940 | down | MANSC domain-containing protein 1 precursor |
| ENSMUSG00000056220 | *PLA2G4A* | 2.70E-03 | -1.0841 | down | cytosolic phospholipase A2 isoform 1 |
| ENSMUSG00000028020 | *GLRB* | 2.54E-06 | -2.0618 | down | glycine receptor subunit beta isoform 4 precursor |
| ENSMUSG00000003585 | *SEC14L2* | 7.37E-05 | -1.9582 | down | SEC14-like protein 2 |
| ENSMUSG00000017390 | *ALDOC* | 2.40E-04 | -1.9107 | down | fructose-bisphosphate aldolase C |
| ENSMUSG00000043003 | *RASEF* | 2.94E-04 | -1.2360 | down | ras and EF-hand domain-containing protein homolog |
| ENSMUSG00000068196 | *COL8A1* | 1.86E-05 | -1.4399 | down | collagen alpha-1(VIII) chain precursor |
| ENSMUSG00000044921 | *RASSF9* | 2.03E-03 | -0.9019 | down | ras association domain-containing protein 9 |
| ENSMUSG00000019810 | *FUCA2* | 3.65E-03 | -0.5880 | down | plasma alpha-L-fucosidase precursor |
| ENSMUSG00000030257 | *SRGAP3* | 6.02E-03 | -1.0804 | down | SLIT-ROBO Rho GTPase-activating protein 3 |
| ENSMUSG00000022900 | *ILDR1* | 3.40E-04 | -1.4818 | down | immunoglobulin-like domain-containing receptor 1 isoform 1 precursor |
| ENSMUSG00000034177 | *RNF43* | 3.99E-03 | -1.3562 | down | E3 ubiquitin-protein ligase RNF43 isoform 1 precursor |
| ENSMUSG00000046352 | *GJB2* | 4.85E-03 | -2.0015 | down | gap junction membrane channel protein beta 2, isoform CRA_b, partial |
| ENSMUSG00000054342 | *KCNN4* | 1.92E-03 | -1.8895 | down | intermediate conductance calcium-activated potassium channel protein 4 |
| ENSMUSG00000028392 | *BSPRY* | 3.61E-03 | -1.6916 | down | B-box and SPRY domain containing, isoform CRA_d, partial |
| ENSMUSG00000027224 | *DUOXA1* | 3.84E-05 | -2.8810 | down | dual oxidase maturation factor 1 isoform X2 [Mus pahari] |
| ENSMUSG00000026321 | *TNFRSF11A* | 2.14E-03 | -1.3088 | down | tumor necrosis factor receptor superfamily member 11A precursor |
| ENSMUSG00000024776 | *STAMBPL1* | 2.28E-03 | -0.7299 | down | AMSH-like protease isoform 1 |
| NewGene_2448 | *NEWGENE_2448* | 7.54E-04 | -2.8038 | down | immunoglobulin light chain variable region, partial |
| ENSMUSG00000026442 | *NFASC* | 5.99E-03 | -1.3573 | down | neurofascin isoform 1 precursor |
| ENSMUSG00000024511 | *RAB27B* | 1.02E-03 | -1.1544 | down | ras-related protein Rab-27B isoform 1 |
| ENSMUSG00000027536 | *CHMP4C* | 8.24E-04 | -1.2198 | down | charged multivesicular body protein 4c |
| ENSMUSG00000032009 | *SESN3* | 7.80E-03 | -0.5867 | down | sestrin-3 |
| ENSMUSG00000038774 | *ASCC3* | 1.02E-03 | -0.6055 | down | activating signal cointegrator 1 complex subunit 3 |
| ENSMUSG00000033268 | *DUOX1* | 1.20E-04 | -3.0634 | down | dual oxidase 1 precursor |
| ENSMUSG00000024066 | *XDH* | 1.08E-03 | -0.6783 | down | xanthine dehydrogenase, isoform CRA_a, partial |
| ENSMUSG00000019876 | *PKIB* | 2.16E-05 | -2.2997 | down | cAMP-dependent protein kinase inhibitor beta isoform X2 |
| ENSMUSG00000032554 | *TRF* | 2.86E-04 | -1.6126 | down | serotransferrin precursor |
| ENSMUSG00000047861 | *FOXI1* | 5.35E-05 | -1.9115 | down | forkhead box protein I1 |
| ENSMUSG00000011034 | *SLC5A1* | 3.25E-04 | -2.0498 | down | sodium/glucose cotransporter 1 |
| ENSMUSG00000061937 | *CSN1S2A* | 5.81E-05 | -7.8400 | down | alpha-S2-casein-like A isoform 1 precursor |
| ENSMUSG00000029304 | *SPP1* | 9.24E-06 | -3.9376 | down | osteopontin isoform 4 precursor |
| ENSMUSG00000042272 | *SESTD1* | 2.30E-03 | -0.5853 | down | SEC14 domain and spectrin repeat-containing protein 1 |
| ENSMUSG00000027966 | *COL11A1* | 6.86E-06 | -1.8290 | down | collagen alpha-1(XI) chain preproprotein |
| ENSMUSG00000048490 | *NRIP1* | 1.80E-03 | -0.6676 | down | nuclear receptor-interacting protein 1 |
| ENSMUSG00000038859 | *BAIAP2L1* | 6.30E-05 | -1.9769 | down | brain-specific angiogenesis inhibitor 1-associated protein 2-like protein 1 |
| ENSMUSG00000020019 | *NTN4* | 9.35E-04 | -0.8037 | down | netrin-4 precursor |
| ENSMUSG00000067006 | *SERPINB5* | 1.64E-03 | -1.1962 | down | serpin B5 isoform a |
| ENSMUSG00000005802 | *SLC30A4* | 8.61E-04 | -0.7351 | down | zinc transporter 4 isoform a |
| ENSMUSG00000033006 | *SOX10* | 2.68E-03 | -1.5565 | down | putative transcription factor, partial |
| ENSMUSG00000040413 | *TIMD2* | 2.76E-05 | -3.1948 | down | T-cell immunoglobulin and mucin domain-containing protein 2 precursor |
| ENSMUSG00000027459 | *FAM110A* | 9.25E-03 | -1.4807 | down | RIKEN cDNA 5430432M24, isoform CRA_d, partial |
| ENSMUSG00000029657 | *HSPH1* | 6.68E-04 | -0.7178 | down | heat shock protein 105 kDa isoform 1 |
| ENSMUSG00000027030 | *STK39* | 4.77E-03 | -0.6358 | down | serine/threonine kinase 39, isoform CRA_a |
| ENSMUSG00000037681 | *ESYT3* | 7.79E-04 | -1.6672 | down | extended synaptotagmin-3 |
| ENSMUSG00000061013 | *MKX* | 5.05E-05 | -2.1733 | down | mohawk, partial |
| ENSMUSG00000029335 | *BMP3* | 1.18E-03 | -0.7990 | down | bone morphogenetic protein 3 isoform 1 precursor |
| NewGene_5769 | *NEWGENE_5769* | 5.07E-03 | -1.8113 | down | immunoglobulin lambda-chain, partial [Mus musculus domesticus] |
| NewGene_2472 | *NEWGENE_2472* | 2.33E-04 | -3.5340 | down | immunoglobulin light chain variable region, partial |
| ENSMUSG00000026110 | *MGAT4A* | 5.58E-03 | -0.7185 | down | alpha-1,3-mannosyl-glycoprotein 4-beta-N-acetylglucosaminyltransferase A |
| ENSMUSG00000000266 | *MID2* | 1.11E-03 | -0.6843 | down | probable E3 ubiquitin-protein ligase MID2 isoform 3 |
| ENSMUSG00000031608 | *GALNT7* | 1.79E-04 | -0.8589 | down | N-acetylgalactosaminyltransferase 7 isoform 1 |
| ENSMUSG00000001827 | *FOLR1* | 1.04E-04 | -3.8652 | down | folate receptor alpha precursor |
| ENSMUSG00000040594 | *RANBP17* | 4.14E-04 | -1.4612 | down | ran-binding protein 17 isoform 1 |
| ENSMUSG00000040728 | *ESRP1* | 8.44E-04 | -1.2110 | down | epithelial splicing regulatory protein 1 isoform 1 |
| ENSMUSG00000026768 | *ITGA8* | 7.67E-06 | -1.7852 | down | integrin alpha-8 preproprotein |
| ENSMUSG00000027315 | *SPINT1* | 4.22E-03 | -0.8709 | down | kunitz-type protease inhibitor 1 precursor |
| ENSMUSG00000042436 | *MFAP4* | 1.43E-03 | -1.6348 | down | microfibril-associated glycoprotein 4 isoform 2 precursor |
| ENSMUSG00000102805 | *GM37240* | 9.04E-03 | -1.2419 | down | arfaptin-1 isoform X2 |
| ENSMUSG00000032532 | *CCK* | 9.08E-03 | -1.7784 | down | cholecystokinin isoform 2 precursor |
| ENSMUSG00000026818 | *CEL* | 1.10E-03 | -4.7162 | down | bile salt-activated lipase precursor |
| ENSMUSG00000067768 | *XLR4B* | 3.69E-03 | -1.7150 | down | X-linked lymphocyte-regulated 4B |
| ENSMUSG00000032068 | *PLET1* | 5.24E-06 | -2.4065 | down | placenta-expressed transcript 1 protein precursor |
| ENSMUSG00000074361 | *C5AR2* | 4.75E-04 | -2.3327 | down | unnamed protein product |
| ENSMUSG00000058638 | *ZFP110* | 5.91E-04 | -0.6010 | down | mCG21533, partial |
| ENSMUSG00000061778 | *MOSPD2* | 2.92E-03 | -0.6115 | down | motile sperm domain-containing protein 2 isoform 2 |
| ENSMUSG00000070702 | *CSN1S1* | 6.71E-05 | -5.7157 | down | alpha-S1-casein isoform a precursor |
| ENSMUSG00000029695 | *AASS* | 6.46E-05 | -2.6138 | down | alpha-aminoadipic semialdehyde synthase, mitochondrial |
| ENSMUSG00000045314 | *SOWAHB* | 3.13E-04 | -2.3646 | down | ankyrin repeat domain-containing protein SOWAHB |
| ENSMUSG00000076434 | *WFDC3* | 2.18E-04 | -2.6455 | down | WAP four-disulfide core domain protein 3 precursor |
| ENSMUSG00000035498 | *CDCP1* | 6.87E-04 | -1.0688 | down | CUB domain-containing protein 1 precursor |
| ENSMUSG00000032528 | *VIPR1* | 1.40E-03 | -0.7967 | down | vasoactive intestinal polypeptide receptor 1 precursor |
| ENSMUSG00000012350 | *EHF* | 4.62E-04 | -1.7404 | down | ETS homologous factor isoform X1 |
| ENSMUSG00000061859 | *PATJ* | 5.94E-05 | -1.1264 | down | inaD-like protein isoform 1 |
| ENSMUSG00000052974 | *CYP2F2* | 8.95E-03 | -0.9380 | down | cytochrome P450 2F2 precursor |
| ENSMUSG00000021638 | *OCLN* | 7.11E-04 | -1.1321 | down | occludin isoform 1 |
| ENSMUSG00000028226 | *MMP16* | 2.27E-04 | -1.7446 | down | matrix metalloproteinase-16 preproprotein |
| ENSMUSG00000022991 | *LALBA* | 3.90E-05 | -4.9353 | down | alpha-lactalbumin precursor |
| ENSMUSG00000027692 | *TNIK* | 1.83E-03 | -0.7792 | down | traf2 and NCK-interacting protein kinase isoform 3 |
| ENSMUSG00000020656 | *GRHL1* | 2.22E-04 | -1.1210 | down | grainyhead-like protein 1 homolog isoform 1 |
| ENSMUSG00000068748 | *PTPRZ1* | 4.00E-03 | -1.0993 | down | receptor-type tyrosine-protein phosphatase zeta isoform 3 precursor |
| ENSMUSG00000038168 | *P3H2* | 7.20E-03 | -0.9769 | down | prolyl 3-hydroxylase 2 precursor |
| ENSMUSG00000044393 | *DSG2* | 3.79E-05 | -1.1703 | down | desmoglein-2 preproprotein |
| ENSMUSG00000022512 | *CLDN1* | 1.93E-05 | -1.6346 | down | claudin-1 |
| ENSMUSG00000033634 | *NAT8F2* | 2.30E-04 | -1.9510 | down | N-acetyltransferase family 8 member 2 |
| ENSMUSG00000022479 | *VDR* | 1.40E-04 | -1.8815 | down | vitamin D3 receptor |
| ENSMUSG00000063157 | *CSN2* | 2.44E-06 | -6.8535 | down | beta-casein isoform a precursor |
| ENSMUSG00000033107 | *RNF125* | 3.03E-04 | -0.9571 | down | E3 ubiquitin-protein ligase RNF125 isoform 2 |
| ENSMUSG00000021553 | *SLC28A3* | 7.39E-07 | -3.0658 | down | solute carrier family 28 member 3 |
| ENSMUSG00000057092 | *FXYD3* | 8.87E-03 | -1.1409 | down | FXYD domain-containing ion transport regulator 3 precursor |
| NewGene_2458 | *NEWGENE_2458* | 1.02E-03 | -2.7718 | down | immunoglobulin light chain variable region, partial |
| ENSMUSG00000024903 | *LAO1* | 4.34E-05 | -6.0769 | down | L-amino acid oxidase 1 precursor |
| ENSMUSG00000048249 | *CREBRF* | 1.56E-03 | -0.6463 | down | CREB3 regulatory factor |
| ENSMUSG00000027111 | *ITGA6* | 1.89E-04 | -0.8285 | down | integrin alpha-6 isoform 1 precursor |
| ENSMUSG00000031278 | *ACSL4* | 1.76E-04 | -0.8757 | down | long-chain-fatty-acid--CoA ligase 4 isoform 1 |
| ENSMUSG00000026070 | *IL18R1* | 3.71E-03 | -1.1112 | down | interleukin-18 receptor 1 isoform a precursor |
| ENSMUSG00000037016 | *FREM2* | 1.44E-03 | -1.2162 | down | FRAS1-related extracellular matrix protein 2 precursor |
| ENSMUSG00000026380 | *TFCP2L1* | 1.03E-03 | -1.5494 | down | transcription factor CP2-like protein 1 |
| NewGene_2447 | *NEWGENE_2447* | 4.59E-03 | -3.2104 | down | mCG141625, partial |
| ENSMUSG00000054169 | *CEACAM10* | 1.37E-04 | -2.5063 | down | carcinoembryonic antigen-related cell adhesion molecule 10 precursor |
| ENSMUSG00000032372 | *PLSCR2* | 6.82E-03 | -1.5599 | down | phospholipid scramblase 2 isoform X1 |
| ENSMUSG00000027996 | *SFRP2* | 2.99E-03 | -1.0214 | down | secreted frizzled-related protein 2 precursor |
| ENSMUSG00000063663 | *BRWD3* | 2.34E-03 | -0.8758 | down | bromodomain and WD repeat-containing protein 3 |
| ENSMUSG00000052271 | *BHLHA15* | 1.25E-03 | -2.4212 | down | class A basic helix-loop-helix protein 15 |
| ENSMUSG00000003849 | *NQO1* | 8.67E-03 | -0.9305 | down | NAD(P)H dehydrogenase [quinone] 1 |
| ENSMUSG00000036882 | *ARHGAP33* | 7.76E-03 | -1.4019 | down | rho GTPase-activating protein 33 isoform a |
| ENSMUSG00000027993 | *TRIM2* | 2.23E-03 | -0.8038 | down | mKIAA0517 protein, partial |
| ENSMUSG00000029787 | *AVL9* | 2.22E-04 | -0.6061 | down | late secretory pathway protein AVL9 homolog |
| ENSMUSG00000030659 | *NUCB2* | 9.78E-04 | -1.0851 | down | unnamed protein product |
| NewGene_2444 | *NEWGENE_2444* | 5.81E-05 | -3.2311 | down | mCG141631, partial |
| ENSMUSG00000054702 | *AP1S3* | 4.45E-03 | -1.3642 | down | AP-1 complex subunit sigma-3 |
| ENSMUSG00000030732 | *CHRDL2* | 1.56E-04 | -5.3087 | down | chordin-like protein 2 isoform 2 precursor |
| ENSMUSG00000021709 | *ERBIN* | 3.20E-04 | -0.6999 | down | mKIAA1225 protein, partial |
| ENSMUSG00000008763 | *MAN1A2* | 9.16E-04 | -0.6117 | down | mannosyl-oligosaccharide 1,2-alpha-mannosidase IB |
| ENSMUSG00000041439 | *MFSD6* | 1.03E-04 | -0.8116 | down | RIKEN cDNA 2210010L05, partial |
| ENSMUSG00000029381 | *SHROOM3* | 3.92E-03 | -1.3775 | down | protein Shroom3 isoform 1 |
| ENSMUSG00000050808 | *MUC15* | 4.54E-03 | -2.9238 | down | mucin-15 isoform a precursor |
| ENSMUSG00000031075 | *ANO1* | 4.06E-03 | -0.9970 | down | anoctamin-1 isoform X13 |
| ENSMUSG00000034435 | *TMEM30B* | 3.09E-03 | -0.9384 | down | cell cycle control protein 50B |
| ENSMUSG00000027186 | *ELF5* | 6.19E-05 | -2.3072 | down | ETS-related transcription factor Elf-5 |
| ENSMUSG00000053877 | *SRCAP* | 8.61E-14 | -2.7608 | down | helicase SRCAP |
| ENSMUSG00000031995 | *ST14* | 9.69E-03 | -1.2569 | down | suppressor of tumorigenicity 14 protein homolog |
| ENSMUSG00000034488 | *EDIL3* | 1.69E-03 | -1.4031 | down | EGF-like repeat and discoidin I-like domain-containing protein 3 precursor |
| ENSMUSG00000000308 | *CKMT1* | 2.16E-03 | -1.5781 | down | creatine kinase, mitochondrial 1, ubiquitous, isoform CRA_c, partial |
| ENSMUSG00000024421 | *LAMA3* | 6.38E-03 | -0.8630 | down | laminin subunit alpha-3 isoform 3B precursor |
| ENSMUSG00000030562 | *NOX4* | 5.02E-06 | -1.6104 | down | NADPH oxidase 4 isoform 1 |
| ENSMUSG00000053461 | *HHIPL2* | 1.98E-06 | -2.9544 | down | HHIP-like protein 2 isoform 1 precursor |
| ENSMUSG00000021696 | *ELOVL7* | 1.83E-06 | -1.7720 | down | elongation of very long chain fatty acids protein 7 |
| ENSMUSG00000026435 | *SLC45A3* | 8.51E-04 | -2.0213 | down | solute carrier family 45 member 3 |
| ENSMUSG00000020644 | *ID2* | 2.97E-03 | -1.1213 | down | DNA-binding protein inhibitor ID-2 |
| ENSMUSG00000051359 | *NCALD* | 3.50E-04 | -1.4841 | down | neurocalcin-delta [Rattus norvegicus] |
| ENSMUSG00000025207 | *SEMA4G* | 6.02E-03 | -1.5595 | down | semaphorin-4G precursor |
| ENSMUSG00000042225 | *AMMECR1* | 5.64E-04 | -0.8461 | down | AMME syndrome candidate gene 1 protein homolog |
| ENSMUSG00000031626 | *SORBS2* | 1.98E-04 | -0.6324 | down | mKIAA0777 protein, partial |
| ENSMUSG00000001672 | *MARVELD3* | 1.76E-03 | -1.3506 | down | MARVEL domain-containing protein 3 isoform a |
| ENSMUSG00000027956 | *TMEM144* | 3.93E-03 | -1.3214 | down | transmembrane protein 144, isoform CRA_a, partial |
| NewGene_2427 | *NEWGENE_2427* | 9.60E-03 | -3.0003 | down | anti-CD25 immunoglobulin light chain variable region, partial |
| ENSMUSG00000028528 | *DNAJC6* | 1.01E-03 | -1.9440 | down | putative tyrosine-protein phosphatase auxilin isoform d |
| ENSMUSG00000000706 | *BTN1A1* | 2.61E-08 | -3.1867 | down | butyrophilin subfamily 1 member A1 precursor |
| NewGene_2479 | *NEWGENE_2479* | 9.18E-03 | -1.8697 | down | mCG131874, partial |
| ENSMUSG00000102758 | *NAALADL2* | 4.59E-05 | -1.7021 | down | inactive N-acetylated-alpha-linked acidic dipeptidase-like protein 2 |
| ENSMUSG00000026090 | *2010300C02RIK* | 8.93E-04 | -1.7278 | down | CRACD-like protein |
| ENSMUSG00000018166 | *ERBB3* | 1.59E-04 | -1.1724 | down | receptor tyrosine-protein kinase erbB-3 precursor |
| ENSMUSG00000093938 | *EVI2B* | 5.83E-05 | -1.7825 | down | protein EVI2B precursor |
| ENSMUSG00000030207 | *FAM234B* | 1.28E-03 | -1.2651 | down | protein FAM234B isoform 2 |
| ENSMUSG00000034586 | *HID1* | 7.09E-03 | -0.8677 | down | RIKEN cDNA C630004H02, isoform CRA_a, partial |
| ENSMUSG00000022949 | *CLIC6* | 1.59E-04 | -1.7981 | down | chloride intracellular channel protein 6 |
| ENSMUSG00000029859 | *EPHA1* | 6.42E-03 | -0.9865 | down | ephrin type-A receptor 1 precursor |
| ENSMUSG00000032358 | *FAM83B* | 7.57E-04 | -1.9029 | down | protein FAM83B |
| ENSMUSG00000029851 | *TCAF2* | 3.88E-04 | -0.8768 | down | TRPM8 channel-associated factor 2 |
| ENSMUSG00000036985 | *ZDHHC9* | 4.21E-03 | -0.7218 | down | palmitoyltransferase ZDHHC9 |
| ENSMUSG00000079293 | *CLEC7A* | 2.86E-03 | -1.1245 | down | Dectin-1, encodes a protein that is part of the C-type lectin domain family |
| ENSMUSG00000060206 | *ZFP462* | 1.86E-03 | -0.7801 | down | zinc finger protein 462 |
| NewGene_4742 | *NEWGENE_4742* | 6.55E-03 | -1.5490 | down | mCG147612, isoform CRA_b, partial |
| ENSMUSG00000025321 | *ITGB8* | 1.82E-04 | -1.4160 | down | integrin beta-8 precursor |
| NewGene_5304 | *NEWGENE_5304* | 6.29E-03 | -1.5555 | down | unnamed protein product |
| ENSMUSG00000051111 | *SV2C* | 1.88E-05 | -2.2012 | down | synaptic vesicle glycoprotein 2C |
| ENSMUSG00000000303 | *CDH1* | 2.11E-03 | -0.8573 | down | cadherin-1 preproprotein |
| ENSMUSG00000007613 | *TGFBR1* | 5.84E-04 | -0.5865 | down | TGF-beta receptor type-1 isoform 1 precursor |
| ENSMUSG00000005125 | *NDRG1* | 1.24E-03 | -1.0298 | down | protein NDRG1 |
| ENSMUSG00000053216 | *BTN2A2* | 2.13E-03 | -1.7210 | down | butyrophilin, subfamily 2, member A2, isoform CRA_b, partial |
| ENSMUSG00000040093 | *BMF* | 1.47E-04 | -1.5852 | down | bcl-2-modifying factor isoform 1 |
| ENSMUSG00000036885 | *ARHGEF26* | 4.88E-03 | -0.6663 | down | rho guanine nucleotide exchange factor 26 |
| ENSMUSG00000029672 | *FAM3C* | 7.85E-03 | -1.0052 | down | DNA segment, Chr 6, Wayne State University 176, expressed |
| ENSMUSG00000034919 | *TTC22* | 6.24E-03 | -1.4329 | down | tetratricopeptide repeat protein 22 |
| ENSMUSG00000027858 | *TSPAN2* | 1.45E-03 | -0.5900 | down | tetraspanin-2 isoform 1 |
| ENSMUSG00000036019 | *TMTC2* | 1.01E-05 | -1.4737 | down | protein O-mannosyl-transferase TMTC2 precursor |
| ENSMUSG00000000216 | *SCNN1G* | 8.36E-05 | -2.7303 | down | amiloride-sensitive sodium channel subunit gamma |
| NewGene_2429 | *NEWGENE_2429* | 2.02E-05 | -2.4293 | down | immunoglobulin light chain variable region, partial |
| ENSMUSG00000020042 | *BTBD11* | 2.43E-03 | -1.4377 | down | ankyrin repeat and BTB/POZ domain-containing protein BTBD11 isoform 1 |
| ENSMUSG00000024781 | *LIPA* | 7.62E-05 | -1.4332 | down | lysosomal acid lipase/cholesteryl ester hydrolase precursor |
| ENSMUSG00000038879 | *NIPAL2* | 5.16E-03 | -1.2250 | down | NIPA-like protein 2 |
| NewGene_4750 | *NEWGENE_4750* | 5.18E-06 | -3.2377 | down | mCG129376 |
| ENSMUSG00000054385 | *CEACAM2* | 8.56E-03 | -1.3681 | down | carcinoembryonic antigen-related cell adhesion molecule 2 isoform b precursor |
| ENSMUSG00000026065 | *SLC9A4* | 2.07E-04 | -2.3547 | down | sodium/hydrogen exchanger 4 precursor |
| ENSMUSG00000019989 | *ENPP3* | 3.79E-06 | -1.3454 | down | ectonucleotide pyrophosphatase/phosphodiesterase family member 3 |
| ENSMUSG00000024544 | *LDLRAD4* | 4.38E-03 | -0.8167 | down | low-density lipoprotein receptor class A domain-containing protein 4 |
| ENSMUSG00000010175 | *PROX1* | 3.05E-06 | -2.2804 | down | prospero homeobox protein 1 isoform 2 |
| NewGene_2405 | *NEWGENE_2405* | 3.52E-05 | -3.0166 | down | mCG130744, partial |
| ENSMUSG00000033799 | *FAM208B* | 1.50E-03 | -0.7581 | down | protein TASOR 2 isoform 1 |
| ENSMUSG00000018849 | *WWC1* | 3.06E-03 | -1.0442 | down | protein KIBRA |
| ENSMUSG00000062995 | *ICA1* | 4.04E-03 | -0.9220 | down | islet cell autoantigen 1 isoform 1 |
| ENSMUSG00000084128 | *ESRP2* | 9.91E-03 | -0.9590 | down | epithelial splicing regulatory protein 2 |
| ENSMUSG00000037145 | *2210407C18RIK* | 3.64E-08 | -3.9580 | down | epithelial progenitor 1 precursor |
| ENSMUSG00000059708 | *AKAP17B* | 3.82E-03 | -0.8722 | down | A-kinase anchor protein 17B |
| ENSMUSG00000021803 | *CDHR1* | 4.19E-10 | -3.4968 | down | cadherin-related family member 1 precursor |
| ENSMUSG00000020181 | *NAV3* | 3.03E-03 | -0.8594 | down | neuron navigator 3 isoform 1 |
| ENSMUSG00000026994 | *GALNT3* | 2.37E-08 | -1.7639 | down | polypeptide N-acetylgalactosaminyltransferase 3 |
| ENSMUSG00000033542 | *ARHGEF5* | 4.07E-05 | -0.8317 | down | rho guanine nucleotide exchange factor 5 |
| ENSMUSG00000029816 | *GPNMB* | 5.90E-03 | -1.1376 | down | transmembrane glycoprotein NMB precursor |
| NewGene_2467 | *NEWGENE_2467* | 2.34E-03 | -2.1175 | down | mCG142182, partial |
| ENSMUSG00000022514 | *IL1RAP* | 4.38E-04 | -0.8816 | down | interleukin 1 receptor accessory protein, isoform CRA_c, partial |
| ENSMUSG00000022865 | *CXADR* | 4.42E-03 | -0.7230 | down | coxsackievirus and adenovirus receptor homolog isoform a precursor |
| ENSMUSG00000091345 | *COL6A5* | 3.28E-03 | -2.3972 | down | collagen alpha-5(VI) chain precursor |
| ENSMUSG00000024268 | *CELF4* | 4.55E-04 | -1.7749 | down | bruno-like 4, RNA binding protein (Drosophila), isoform CRA_c, partial |
| ENSMUSG00000026961 | *LRRC26* | 9.57E-03 | -1.9251 | down | leucine-rich repeat-containing protein 26 precursor |
| ENSMUSG00000033161 | *ATP1A1* | 6.89E-03 | -0.6715 | down | sodium/potassium-transporting ATPase subunit alpha-1 |
| ENSMUSG00000026077 | *NPAS2* | 6.82E-04 | -2.4679 | down | neuronal PAS domain-containing protein 2 |
| ENSMUSG00000031488 | *RAB11FIP1* | 3.59E-04 | -1.2088 | down | rab11 family-interacting protein 1 isoform 1 |
| ENSMUSG00000053687 | *DPEP2* | 6.41E-03 | 1.8398 | up | dipeptidase 2 isoform X1 |
| ENSMUSG00000032014 | *OAF* | 5.18E-03 | 0.7701 | up | out at first protein homolog precursor |
| ENSMUSG00000047656 | *TRPT1* | 4.76E-03 | 0.6992 | up | tRNA 2'-phosphotransferase 1 |
| ENSMUSG00000001739 | *CLDN15* | 5.49E-03 | 0.6388 | up | claudin-15 |
| ENSMUSG00000089665 | *FCOR* | 2.94E-03 | 0.8943 | up | foxo1-corepressor |
| ENSMUSG00000060950 | *TRMT61A* | 2.31E-03 | 0.6367 | up | tRNA (adenine(58)-N(1))-methyltransferase catalytic subunit TRMT61A |
| ENSMUSG00000022555 | *DGAT1* | 2.89E-06 | 0.9637 | up | diacylglycerol O-acyltransferase 1 |
| ENSMUSG00000029570 | *LFNG* | 8.32E-04 | 0.9190 | up | beta-1,3-N-acetylglucosaminyltransferase lunatic fringe precursor |
| ENSMUSG00000032172 | *OLFM2* | 4.67E-03 | 1.0398 | up | noelin-2 isoform 1 |
| ENSMUSG00000002059 | *RAB34* | 2.37E-06 | 0.7103 | up | ras-related protein Rab-34 |
| ENSMUSG00000018169 | *MFNG* | 1.58E-03 | 0.6753 | up | beta-1,3-N-acetylglucosaminyltransferase manic fringe |
| ENSMUSG00000074604 | *MGST2* | 5.20E-04 | 0.9820 | up | microsomal glutathione S-transferase 2 isoform 1 |
| ENSMUSG00000021453 | *GADD45G* | 4.72E-07 | 1.0861 | up | growth arrest and DNA damage-inducible protein GADD45 gamma |
| ENSMUSG00000109293 | *DCST2* | 6.92E-03 | 0.7915 | up | DC-STAMP domain-containing protein 2 |
| ENSMUSG00000030731 | *SYT3* | 1.62E-03 | 0.8664 | up | synaptotagmin-3 |
| ENSMUSG00000031808 | *SLC27A1* | 3.72E-08 | 1.1229 | up | solute carrier family 27 (fatty acid transporter), member 1, isoform CRA_b |
| ENSMUSG00000020131 | *PCSK4* | 1.12E-04 | 1.4614 | up | proprotein convertase subtilisin/kexin type 4 precursor |
| ENSMUSG00000053080 | *2700081O15RIK* | 4.28E-03 | 0.5974 | up | uncharacterized protein C11orf95 homolog |
| ENSMUSG00000038400 | *PMEPA1* | 5.63E-04 | 0.6752 | up | Pmepa1 protein, partial |
| ENSMUSG00000056899 | *IMMP2L* | 9.13E-03 | 0.6332 | up | mitochondrial inner membrane protease subunit 2 |
| ENSMUSG00000028064 | *SEMA4A* | 3.10E-04 | 0.7302 | up | semaphorin-4A isoform X2 |
| ENSMUSG00000073802 | *CDKN2B* | 2.83E-04 | 0.8711 | up | cyclin-dependent kinase 4 inhibitor B |
| ENSMUSG00000045777 | *IFITM10* | 1.99E-03 | 0.9555 | up | interferon-induced transmembrane protein 10 isoform X1 |
| ENSMUSG00000078656 | *VPS25* | 7.53E-03 | 0.6576 | up | vacuolar protein-sorting-associated protein 25 isoform 3 |
| ENSMUSG00000000120 | *NGFR* | 5.57E-03 | 1.9554 | up | tumor necrosis factor receptor superfamily member 16 precursor |
| ENSMUSG00000032643 | *FHL3* | 1.73E-04 | 0.7841 | up | four and a half LIM domains protein 3 |
| ENSMUSG00000028393 | *ALAD* | 6.44E-03 | 0.6089 | up | delta-aminolevulinic acid dehydratase |
| ENSMUSG00000069917 | *HBA-A2* | 9.64E-03 | 0.9020 | up | hemoglobin alpha, adult chain 2 |
| ENSMUSG00000016194 | *HSD11B1* | 3.02E-03 | 0.7081 | up | hydroxysteroid 11-beta dehydrogenase 1, partial |
| ENSMUSG00000061024 | *RRS1* | 3.21E-03 | 0.6009 | up | mKIAA0112 protein, partial |
| ENSMUSG00000095789 | *NUPR1L* | 2.43E-04 | 1.3957 | up | nuclear protein 2 |
| ENSMUSG00000010307 | *TMEM86A* | 5.53E-04 | 1.4862 | up | lysoplasmalogenase-like protein TMEM86A |
| ENSMUSG00000048772 | *TMEM53* | 5.64E-03 | 0.7507 | up | transmembrane protein 53 isoform b |
| ENSMUSG00000039956 | *MRAP* | 5.65E-03 | 0.6870 | up | melanocortin-2 receptor accessory protein |
| ENSMUSG00000038150 | *ORMDL3* | 3.01E-03 | 0.6082 | up | ORM1-like protein 3 |
| ENSMUSG00000030086 | *CHCHD6* | 2.61E-04 | 0.6676 | up | MICOS complex subunit Mic25 isoform 1 |
| ENSMUSG00000114635 | *GM49392* | 7.21E-03 | 1.0658 | up | protein CTLA-2-beta isoform a |
| ENSMUSG00000078139 | *AK157302* | 1.11E-03 | 0.9829 | up | mCG50313 |
| ENSMUSG00000028076 | *CD1D1* | 6.63E-04 | 0.7615 | up | antigen-presenting glycoprotein CD1d1 isoform 1 precursor |
| ENSMUSG00000031722 | *HP* | 9.27E-03 | 0.6581 | up | haptoglobin isoform 1 preproprotein |
| ENSMUSG00000005057 | *SH2B2* | 6.55E-03 | 0.6636 | up | SH2B adaptor protein 2, isoform CRA_c, partial |
| ENSMUSG00000079444 | *GM21981* | 5.63E-03 | 0.5863 | up | prickle-like protein 4 |
| ENSMUSG00000022843 | *CLCN2* | 9.68E-03 | 0.8659 | up | chloride channel protein 2 |
| ENSMUSG00000063931 | *PEPD* | 1.05E-05 | 0.8689 | up | xaa-Pro dipeptidase |
| ENSMUSG00000031821 | *GINS2* | 1.43E-06 | 1.1218 | up | DNA replication complex GINS protein PSF2 |
| ENSMUSG00000002346 | *SLC25A42* | 9.56E-03 | 1.1313 | up | mitochondrial coenzyme A transporter SLC25A42 |
| ENSMUSG00000028789 | *AZIN2* | 9.75E-03 | 0.7282 | up | antizyme inhibitor 2 isoform 1 |
| ENSMUSG00000043122 | *A530016L24RIK* | 6.08E-03 | 0.7948 | up | nutritionally-regulated adipose and cardiac-enriched protein |
| ENSMUSG00000018339 | *GPX3* | 3.37E-03 | 0.9051 | up | glutathione peroxidase 3 [Mus caroli] |
| ENSMUSG00000020610 | *AMZ2* | 6.85E-03 | 0.9970 | up | archaemetzincin-2 |
| ENSMUSG00000009394 | *SYN2* | 4.27E-03 | 1.6855 | up | synapsin-2 isoform IIb |
| ENSMUSG00000035513 | *NTNG2* | 4.49E-03 | 0.6532 | up | netrin-G2 isoform c precursor |
| ENSMUSG00000086962 | *GM12248* | 2.96E-03 | 2.5378 | up | fatty acid hydroxylase domain-containing protein 2 |
| ENSMUSG00000050896 | *RTN4RL2* | 3.76E-03 | 0.9683 | up | reticulon-4 receptor-like 2 precursor |
| ENSMUSG00000057054 | *INCA1* | 3.15E-04 | 0.8998 | up | protein INCA1 isoform 1 |
| ENSMUSG00000039533 | *MMD2* | 2.48E-08 | 3.5132 | up | monocyte to macrophage differentiation factor 2 |
| ENSMUSG00000073940 | *HBB-BT* | 8.08E-03 | 1.1198 | up | hemoglobin, beta adult s chain |
| ENSMUSG00000000531 | *GRASP* | 2.29E-03 | 0.6390 | up | general receptor for phosphoinositides 1-associated scaffold protein |
| ENSMUSG00000035283 | *ADRB1* | 1.40E-03 | 0.7802 | up | beta-1 adrenergic receptor |
| ENSMUSG00000042745 | *ID1* | 2.13E-03 | 0.8072 | up | inhibitor of DNA binding 1, isoform CRA_b, partial |
| ENSMUSG00000031762 | *MT2* | 1.44E-04 | 0.9265 | up | metallothionein-2 |
| ENSMUSG00000046215 | *RPRML* | 1.02E-06 | 1.4154 | up | reprimo-like protein |
| ENSMUSG00000006958 | *CHRD* | 7.45E-03 | 0.7173 | up | chordin isoform X6 |
| ENSMUSG00000049580 | *TSKU* | 1.19E-03 | 1.0152 | up | unnamed protein product, partial |
| ENSMUSG00000020086 | *H2AFY2* | 7.96E-04 | 0.7776 | up | core histone macro-H2A.2 [Rattus norvegicus] |
| ENSMUSG00000029449 | *RHOF* | 3.42E-04 | 0.8362 | up | rho-related GTP-binding protein RhoF |
| ENSMUSG00000095562 | *GM21887* | 9.93E-03 | 1.3521 | up | erythroid differentiation regulator |
| ENSMUSG00000041000 | *TRIM62* | 1.53E-03 | 1.0373 | up | E3 ubiquitin-protein ligase TRIM62 isoform 2 |
| ENSMUSG00000039114 | *NRN1* | 1.80E-03 | 1.0398 | up | neuritin isoform 2 |
| ENSMUSG00000035172 | *PLEKHH3* | 3.38E-04 | 0.9451 | up | pleckstrin homology domain-containing family H member 3 precursor |
| ENSMUSG00000036111 | *LMO1* | 1.88E-03 | 0.7157 | up | rhombotin-1 isoform 1 |
| ENSMUSG00000027950 | *CHRNB2* | 3.04E-03 | 1.0877 | up | neuronal acetylcholine receptor subunit beta-2 precursor |
| ENSMUSG00000024968 | *RCOR2* | 3.10E-04 | 1.0856 | up | REST corepressor 2 |
| ENSMUSG00000023067 | *CDKN1A* | 1.50E-03 | 0.8224 | up | cyclin-dependent kinase inhibitor 1 |
| ENSMUSG00000022018 | *RGCC* | 9.97E-06 | 0.8343 | up | regulator of cell cycle RGCC |
| ENSMUSG00000024962 | *VEGFB* | 8.83E-03 | 0.6247 | up | vascular endothelial growth factor B isoform Vegf-b186 precursor |
| ENSMUSG00000003378 | *GRIK5* | 9.35E-03 | 0.6918 | up | glutamate receptor ionotropic, kainate 5 [Arvicanthis niloticus] |
| ENSMUSG00000034957 | *CEBPA* | 8.17E-07 | 0.8312 | up | CCAAT/enhancer-binding protein alpha isoform a |
| ENSMUSG00000063953 | *AMD2* | 3.99E-04 | 1.2788 | up | S-adenosylmethionine decarboxylase proenzyme 2 |
| ENSMUSG00000018907 | *ALOX12E* | 2.10E-03 | 2.4357 | up | polyunsaturated fatty acid (12S)/(13S)-lipoxygenase, isoform X1 |
| ENSMUSG00000068758 | *IL3RA* | 4.07E-04 | 0.8207 | up | interleukin-3 receptor subunit alpha isoform X5 |
| ENSMUSG00000042569 | *DHRS7B* | 4.15E-04 | 0.7119 | up | dehydrogenase/reductase SDR family member 7B isoform X1 |
| ENSMUSG00000056487 | *METTL7A2* | 2.90E-04 | 1.4918 | up | methyltransferase like 7A2 |
| ENSMUSG00000037664 | *CDKN1C* | 2.72E-05 | 0.7697 | up | cyclin-dependent kinase inhibitor 1C isoform 1 |
| ENSMUSG00000044405 | *ADIG* | 1.93E-04 | 0.9620 | up | adipogenin |
| ENSMUSG00000030284 | *CRELD1* | 2.66E-04 | 0.6482 | up | protein disulfide isomerase Creld1 precursor |
| ENSMUSG00000052934 | *FBXO31* | 4.41E-04 | 0.5936 | up | F-box only protein 31 |
| ENSMUSG00000008035 | *MID1IP1* | 8.35E-03 | 0.6847 | up | mid1-interacting protein 1 |
| ENSMUSG00000007805 | *TWIST2* | 4.98E-03 | 0.7597 | up | a basic helix-loop-helix (bHLH) transcription factor |
| ENSMUSG00000015843 | *RXRG* | 5.25E-03 | 0.6235 | up | retinoic acid receptor RXR-gamma isoform 1 |
| ENSMUSG00000044927 | *H1FX* | 5.64E-05 | 2.1381 | up | H1 histone family, member X |
| ENSMUSG00000054434 | *TMEM120B* | 4.57E-05 | 0.7587 | up | transmembrane protein 120B |
| ENSMUSG00000033088 | *TRIOBP* | 1.80E-04 | 0.6126 | up | TRIO and F-actin-binding protein isoform 3 |
| ENSMUSG00000039886 | *TMEM120A* | 6.93E-04 | 1.1053 | up | ion channel TACAN |
| ENSMUSG00000049892 | *RASD1* | 1.39E-05 | 1.6156 | up | dexamethasone-induced Ras-related protein 1 [Arvicanthis niloticus] |
| ENSMUSG00000071497 | *NUTF2-PS1* | 9.24E-05 | 1.5730 | up | nuclear transport factor 2 isoform X2 [Castor canadensis] |
| ENSMUSG00000059824 | *DBP* | 2.42E-04 | 1.5917 | up | D site-binding protein |
| ENSMUSG00000038236 | *HOXA7* | 2.47E-03 | 0.7771 | up | homeobox A7, isoform CRA_d |
| ENSMUSG00000045377 | *TMEM88* | 1.27E-03 | 0.7690 | up | transmembrane protein 88 |
| ENSMUSG00000072624 | *GM5460* | 1.10E-03 | 1.7682 | up | uncharacterized protein LOC432838 isoform X2 |
| ENSMUSG00000034664 | *ITGA2B* | 5.66E-04 | 1.3133 | up | integrin alpha-IIb precursor |
| ENSMUSG00000056938 | *ACBD4* | 8.31E-03 | 0.6163 | up | acyl-CoA-binding domain-containing protein 4 isoform X4 [Mus caroli] |
| NewGene_595 | *NEWGENE_595* | 2.24E-03 | 0.7175 | up | unnamed protein product |
| ENSMUSG00000041801 | *PHLDA3* | 2.59E-03 | 0.7165 | up | pleckstrin homology-like domain family A member 3 |
| ENSMUSG00000071657 | *BSCL2* | 2.59E-04 | 0.6501 | up | seipin isoform 1 |
