# Supplementary material for: Metformin reduces basal subpopulation and attenuates mammary epithelial cell stemness in FVB/N mice
Source: Front Cell Dev Biol. 2024 Jul 11;12:1427395. doi: 10.3389/fcell.2024.1427395 (PMC11269140; doi:10.3389/fcell.2024.1427395)
Supplement: Supplementary file 2 [file DataSheet1.docx]

Supplementary Material

**Supplementary Figure 1.** **Densitometry analysis of protein levels of markers in the AMPK, RTK, ER, and β-Catenin pathways in mammary tissues of control and metformin-treated FVB/N mice.** Based on the Western blot data in Fig. 4, signals of individual markers in the control and metformin-treated groups were scanned. The relative signals of indicated markers were quantified with ImageJ software, which were normalized with the signals of loading control. For the phosphorylated proteins, the relative activation/phosphorylation of each marker was further normalized with corresponding total protein signals. (* p < 0.05, ** p < 0.01, NS = no significance)

**Supplementary Figure 2. Protein levels of total and phosphorylated mTOR and S6 in mammary and liver tissues of FVB/N mice.** Protein lysates of mammary and liver tissues were extracted from 10-week-old female FVB/N mice followed by Western blot analysis.

**Supplemental Fig. 3C**


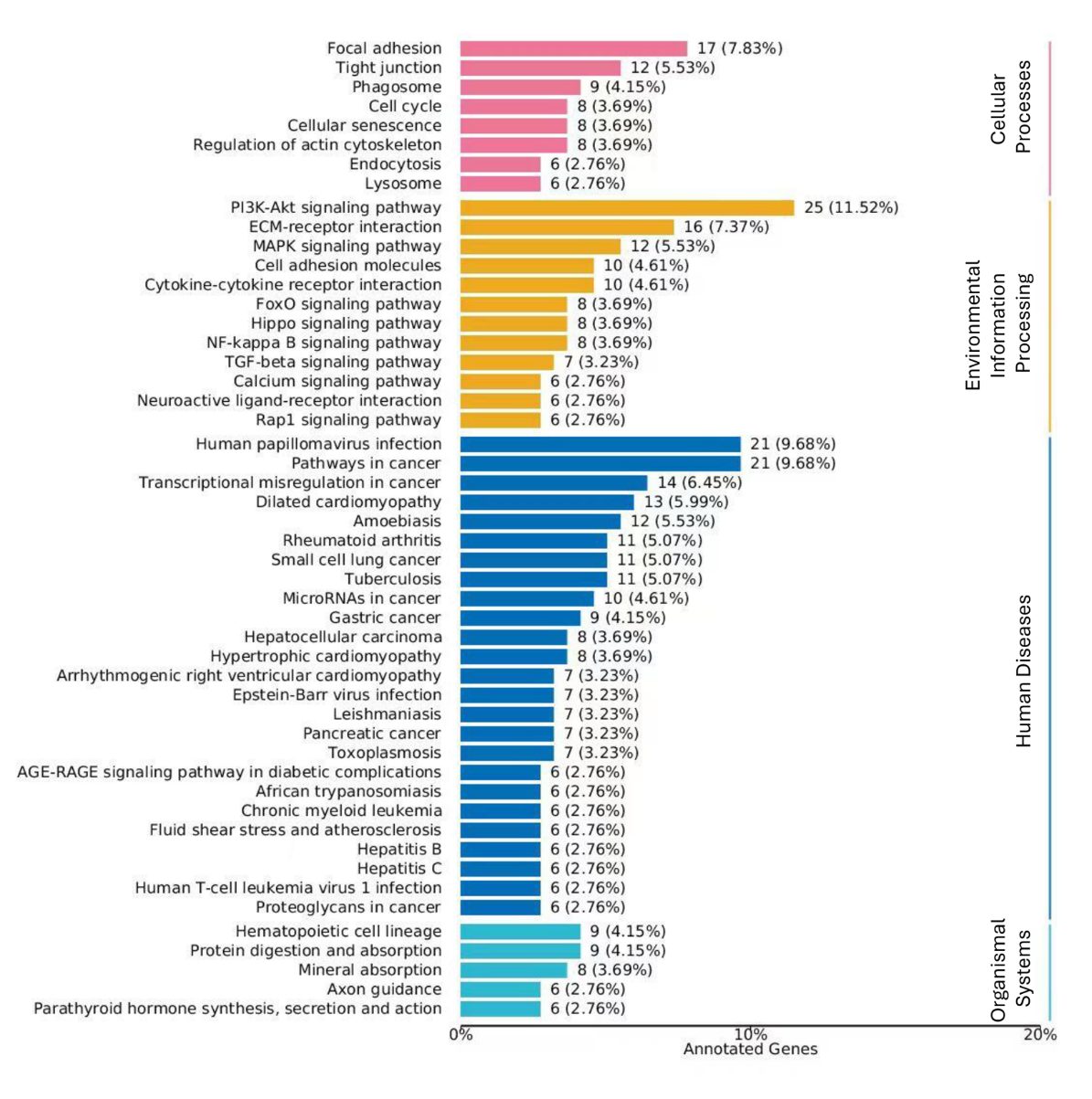


**Supplementary Figure 3. KEGG pathway enrichment analysis of DEGs in three different ways.** In contrast to Fig. 7, which presents the top 20 pathways based on both upregulated and downregulated differentially expressed genes (DEGs), the KEGG pathway analysis in this figure was performed separately based on downregulated DEGs (Fig. 3A), upregulated DEGs (Fig. 3B) and total DEGs (Fig. 3C).
